# *WRKY1* acts as a key component improving resistance against *Alternaria solani* in wild tomato*, Solanum arcanum* Peralta

**DOI:** 10.1111/pbi.12892

**Published:** 2018-05-24

**Authors:** Balkrishna A. Shinde, Bhushan B. Dholakia, Khalid Hussain, Asaph Aharoni, Ashok P. Giri, Avinash C. Kamble

**Affiliations:** ^1^ Department of Botany Savitribai Phule Pune University (Formerly University of Pune) Pune India; ^2^ Division of Biochemical Sciences Plant Molecular Biology Unit CSIR‐National Chemical Laboratory Pune India; ^3^ Department of Plant and Environmental Sciences Weizmann Institute of Science Rehovot Israel

**Keywords:** *Alternaria solani*, early blight, *Solanum arcanum*, Tomato, *WRKY1*, *XTH5*

## Abstract

Early blight (EB), caused by *Alternaria solani,* is a major threat to global tomato production. In comparison with cultivated tomato (*Solanum lycopersicum*), a wild relative, *S. arcanum* exhibits strong resistance against EB. However, molecular cascades operating during EB resistance in wild or cultivated tomato plants are largely obscure. Here, we provide novel insight into spatio‐temporal molecular events in *S. arcanum* against *A. solani*. Transcriptome and co‐expression analysis presented 33‐*WRKY*s as promising candidates of which 12 *SaWRKY*s displayed differential expression patterns in resistant and susceptible accessions during EB disease progression. Among these, *SaWRKY1* exhibited induced expression with significant modulation in xyloglucan endotrans hydrolase 5 (*XTH5*) and *MYB2* expressions that correlated with the disease phenotypes. Electro‐mobility shift assay confirmed physical interaction of recombinant SaWRKY1 to *SaXTH5* and *SaMYB2* promoters. Comparative *WRKY1* promoter analysis between resistant and susceptible plants revealed the presence of crucial motifs for defence mechanism exclusively in resistant accession. Additionally, many defence‐related genes displayed significant expression variations in both the accessions. Further, *WRKY1* overexpressing transgenic plants exhibited higher levels of EB resistance while RNAi silencing lines had increased susceptibility to *A. solani* with altered expression of *XTH5* and *MYB2*. Overall, these findings demonstrate the positive influence of *WRKY1* in improving EB resistance in wild tomato and this could be further utilized as a potential target through genetic engineering to augment protection against *A. solani* in crop plants.

## Introduction

Plants have evolved diverse defence mechanisms to protect themselves against pathogen attack. Rapidly activated defence responses are mediated by complex signalling pathways affecting numerous cellular and molecular processes that lead to resistance. These include generation of reactive oxygen species, cell wall lignification, accumulation of antimicrobial compounds and activation of defence‐related genes (Durrant and Dong, 2004; Hammond‐kosack and Jones, 1996; Jones and Dangl, 2006; Seo and Choi, 2015). These host responses to pathogen invasion are mediated by phytohormones such as salicylic acid (SA), jasmonic acid (JA) and ethylene (ET) (Bakshi and Oelmuller, 2014; Pieterse *et al*., 2012). This in turn may help the plant to fine‐tune the regulation of particular defence pathway and provide optimal protection (Eulgem, 2005; Pieterse *et al*., 2001; Rushton *et al*., 2010). Therefore, to understand the plant defence response, it is important to identify key regulatory factors. A number of transcription factors (TFs) orchestrating signal crosstalk have been identified in *Arabidopsis* and other plants. These TFs are involved in activation or inhibition of target genes alone or via interactions with other proteins (Singh *et al*., 2002; Tsuda and Somssich, 2015). Among these, WRKYs are one of the most important TFs playing role in both abiotic and biotic stresses (Bakshi and Oelmuller, 2014; Pandey and Somssich, 2009; Ulker and Somssich, 2004). WRKY proteins interact with W‐boxes located in the promoter regions of several plant defence genes (Bakshi and Oelmuller, 2014; Ulker and Somssich, 2004). These boxes are found in clusters within the short stretches in promoters indicating potential synergistic interactions of different WRKYs (Dong *et al*., 2003; Eulgem *et al*., 1999; Eulgem and Somssich, 2007).

Tomato (*Solanum lycopersicum* L.) is the most important global vegetable crop; however, its production is severally affected due to biotic stresses including fungi, bacteria, viruses, insects and nematodes. Therefore, breeding tomato for disease resistance is one of the current critical necessities. Early blight (EB) is one of the most economically important tomato disease caused by *Alternaria solani* Jones and Grout, a foliar necrotrophic pathogen. Although EB resistance is not known to exist in cultivated tomato, robust resistance against *A. solani* has been identified in some of the wild relatives (Chaerani *et al*., 2007; Foolad *et al*., 2000). Significant modulations in secondary metabolites have been recently identified during wild tomato*–A. solani* interactions (Shinde *et al*., 2017). However, molecular cascades operating during EB resistance in such wild tomato against *A. solani* are not fully understood. Here, we developed co‐expression networks of potential *WRKY* targets and identified differential expression of *WRKY* genes in susceptible and resistant *S. arcanum* Peralta accessions upon *A. solani* inoculation at different stages of disease progression. Further, functional characterization with transgenic plants indicated that *WRKY1* could influence the EB defence in tomato.

## Results

### Co‐expression analysis created networks of *WRKY*‐targeted genes

**Figure 1:**
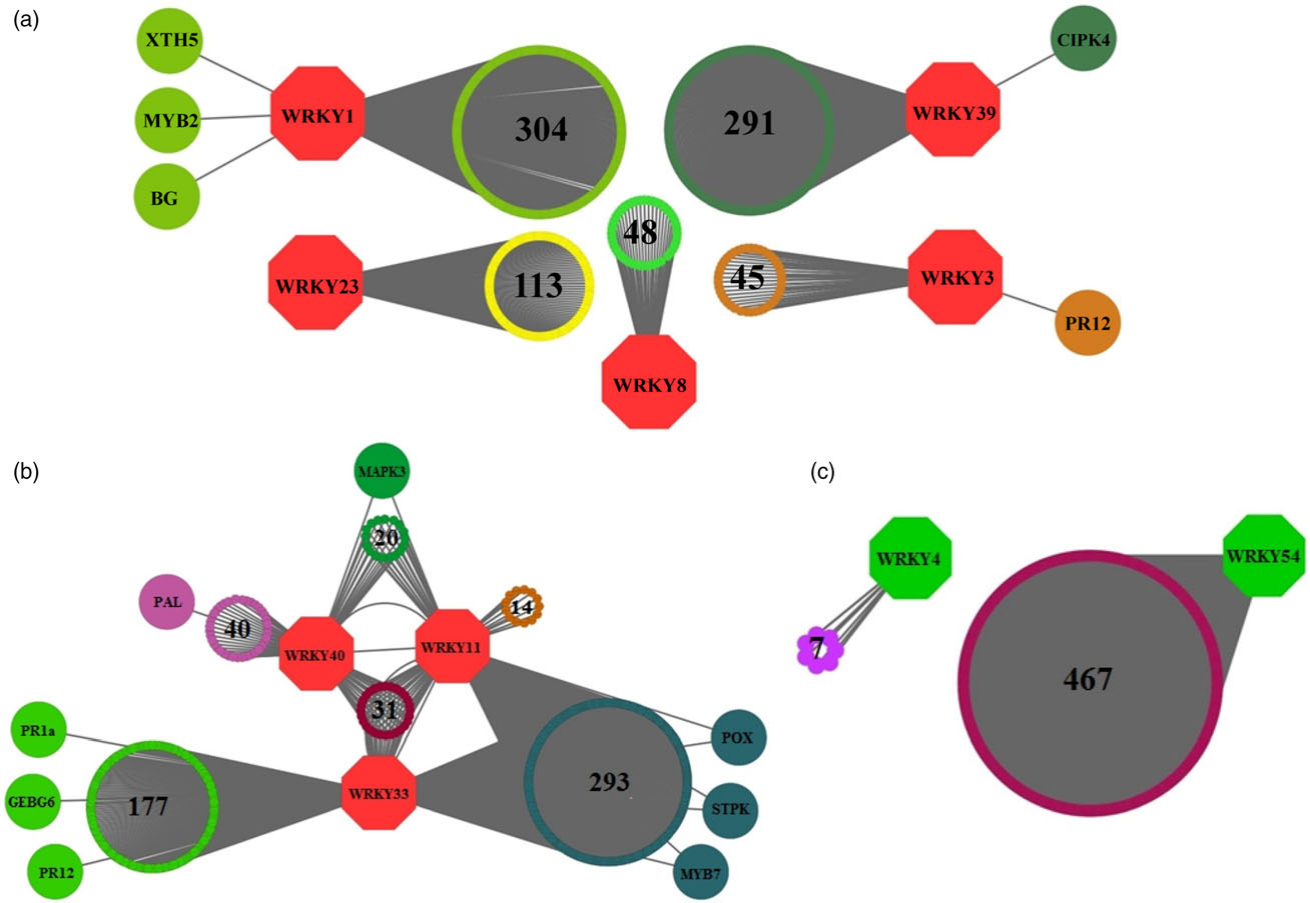
*WRKY* Co‐expression network analysis using Cytoscape 3.1.1. Co‐expressed genes for ‘baits’ from tomato, group A–*WRKY* genes (*SlWRKY1, SlWRKY3, SlWRKY8, SlWRKY23* and *SlWRKY39*) (a), group B–*WRKY* genes (*SlWRKY11, SlWRKY33 and SlWRKY40*) (b) and group C–*WRKY* genes (*SlWRKY4 and SlWRKY54*) (c). Continuous (*r*‐value > 0.8) lines connect co‐expressed genes.

Considering *S. arcanum* closely related to cultivated tomato, co‐expression analysis was performed using available *S. lycopersicum* transcriptomic data (Itkin *et al*., 2011) to find potential *WRKY*s and their downstream targets. Based on the literature and experimental evidences for the *WRKYs* which are playing role in plant defence, 33 *WRKYs* were selected out of 81 in preliminary bioinformatic analysis. Of these, 10 *SlWRKY*s displayed 1389 co‐expressed genes (CEG), while *SlWRKY19* and *SlWRKY22* did not show any CEG with *r *≤* *0.8 (Figure 1; Table [Link], [Link], [Link]). To understand biological role of these CEG, gene ontology (GO) annotations were performed that yielded 4984 GO annotations, representing diverse functions, processes or components. From 886 CEG with significant GO terms, 306 genes indicated GO terms related to defence [i.e. response to stimulus (156 genes), immune system process (35 genes) and biological regulation (114 genes)] (Figure S1; Table S4). After removal of duplication, unique CEG were further analysed to find 5′ *cis*‐regulatory elements in their promoter regions.

### High frequency of W‐box in CEG indicated promising *WRKY* targets

Promoter region of 186 unique CEG and eight known tomato defence genes depicted several 5′ *cis*‐acting regulatory elements involved in phytohormones and biotic stress regulation. Core elements such as TATA, CAAT and CCAAT were located closest to the translational start site (TSS) (ATG). We selected the 5′ *cis*‐regulatory elements related to biotic stress including ERE, GCC, GT1 and W‐boxes (Table S5) to better understand host response during EB disease. Interestingly, promoters of all the unique defence genes revealed high frequency of GT1 box, which is crucial against pathogen response. In few defence genes, W‐boxes were distributed in proximal region while others were in distal region. Around 60% of them revealed high frequency of W‐boxes (Table S6) and were further utilized for expression variations in mock‐ and pathogen‐treated plants using qRT‐PCR.

### Contrasting expression patterns of *SaWRKY*s upon *A. solani* inoculation

**Figure 2:**
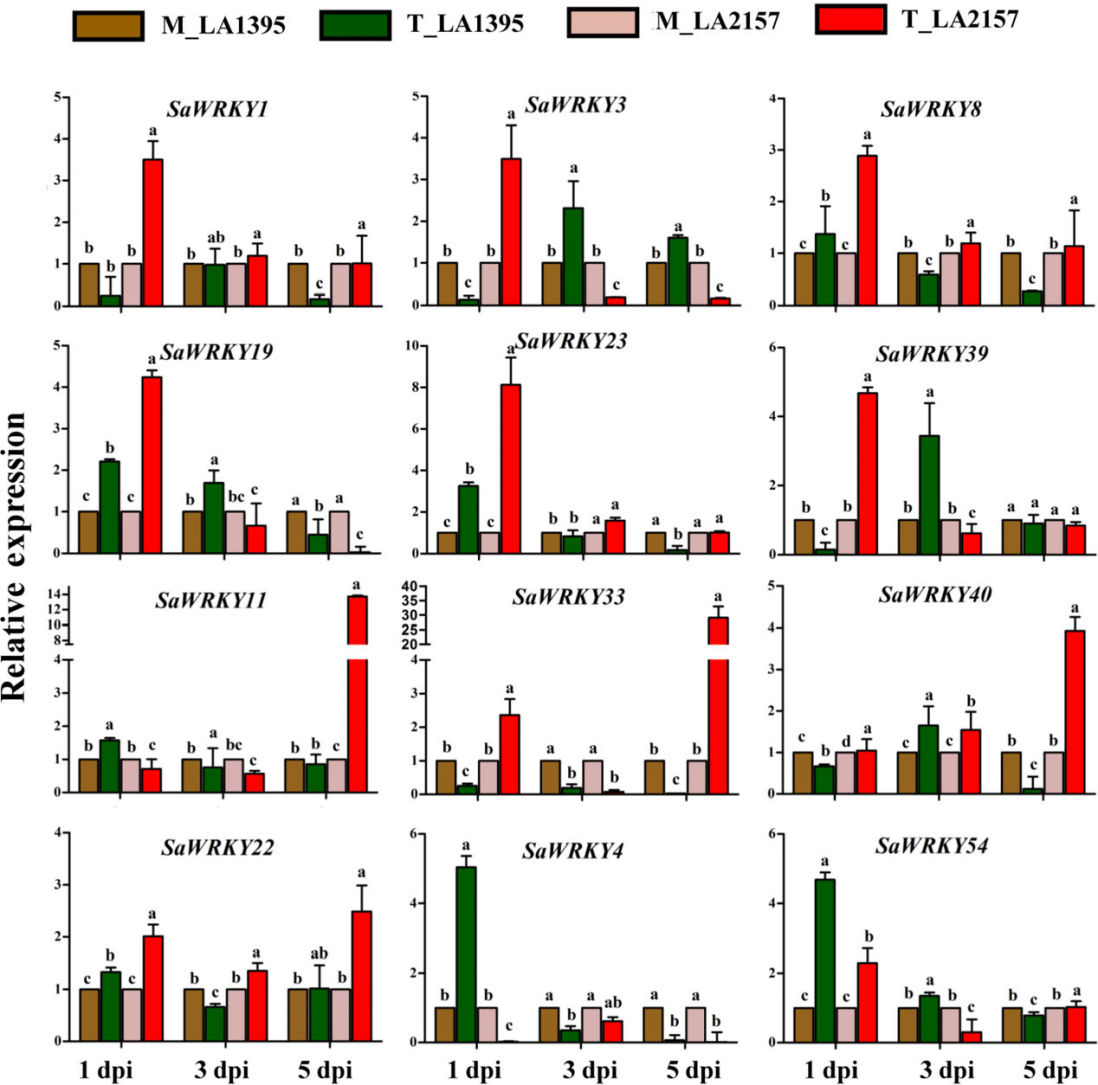
Expression profiles of *WRKY* genes in susceptible (LA1395) and resistant (LA2157) *Solanum arcanum* accessions. Inoculated with *Alternaria solani* spore suspension (3–4 × 10^3^ spores/mL), leaf samples were collected at 1, 3, and 5 dpi. qRT‐PCR was carried out with *SlEF1α* gene as internal control, and expression was normalized to the corresponding mock (M)‐ and *A. solani* spore‐inoculated (T) samples. The values represent means ± SE of three biological replicates each with three technical replicates. Bars represent the standard errors of the means. Different letters indicate significant differences according to Duncan's test (*P *<* *0.05). Similar pattern of gene expression was obtained in two independent experiments. dpi, days post‐inoculation; M, mock inoculated, T, inoculated with *A. solani* spores; Sa, *S. arcanum* Peralta.

EB disease severity on leaves of LA2157 (resistant) and LA1395 (susceptible) *S. arcanum* accessions was assessed as percentage disease index (PDI) after 1, 3 and 5 days postinoculation (dpi) of *A. solani* along with mock (water‐treated) plants (Shinde *et al*., 2017). EB lesions on leaf area progressed rapidly in susceptible compared to resistant plants at all the time points, suggesting LA1395 was susceptible (henceforth, S) while LA2157 had effective resistance (henceforth, R). Experimental validation of above‐mentioned 12 *WRKYs* showed significant differential expression between pathogen‐inoculated R and S accessions during EB disease progression. Expression levels of *SaWRKY1, SaWRKY3, SaWRKY8, SaWRKY19, SaWRKY23* and *SaWRKY39* were significantly increased (up to 3.5‐, 3.5‐, 2.9‐, 4.2‐, 8.1‐ and 4.7‐fold, respectively) in R as compared to S plants at 1 dpi (Figure 2). While expression of *SaWRKY11* (up to 13.7‐fold)*, SaWRKY33* (up to 29‐fold) and *SaWRKY40* (up to 3.9‐fold) displayed significant up‐regulation at 5 dpi, they remained unchanged in S accession at 1 and 3 dpi. Additionally, *SaWRKY22* revealed higher expression (up to 2.4‐fold) in R plants at all the time points. On the other hand, *SaWRKY4* and *SaWRKY54* exhibited significantly elevated (up to 5‐fold and 4.7‐fold, respectively) levels in S accession at 1 dpi and remained unchanged in both the accessions at 3 and 5 dpi after *A. solani* inoculation (Figure 2).

### High expression of co‐expressed defence genes and effect of SA during EB defence

**Figure 3:**
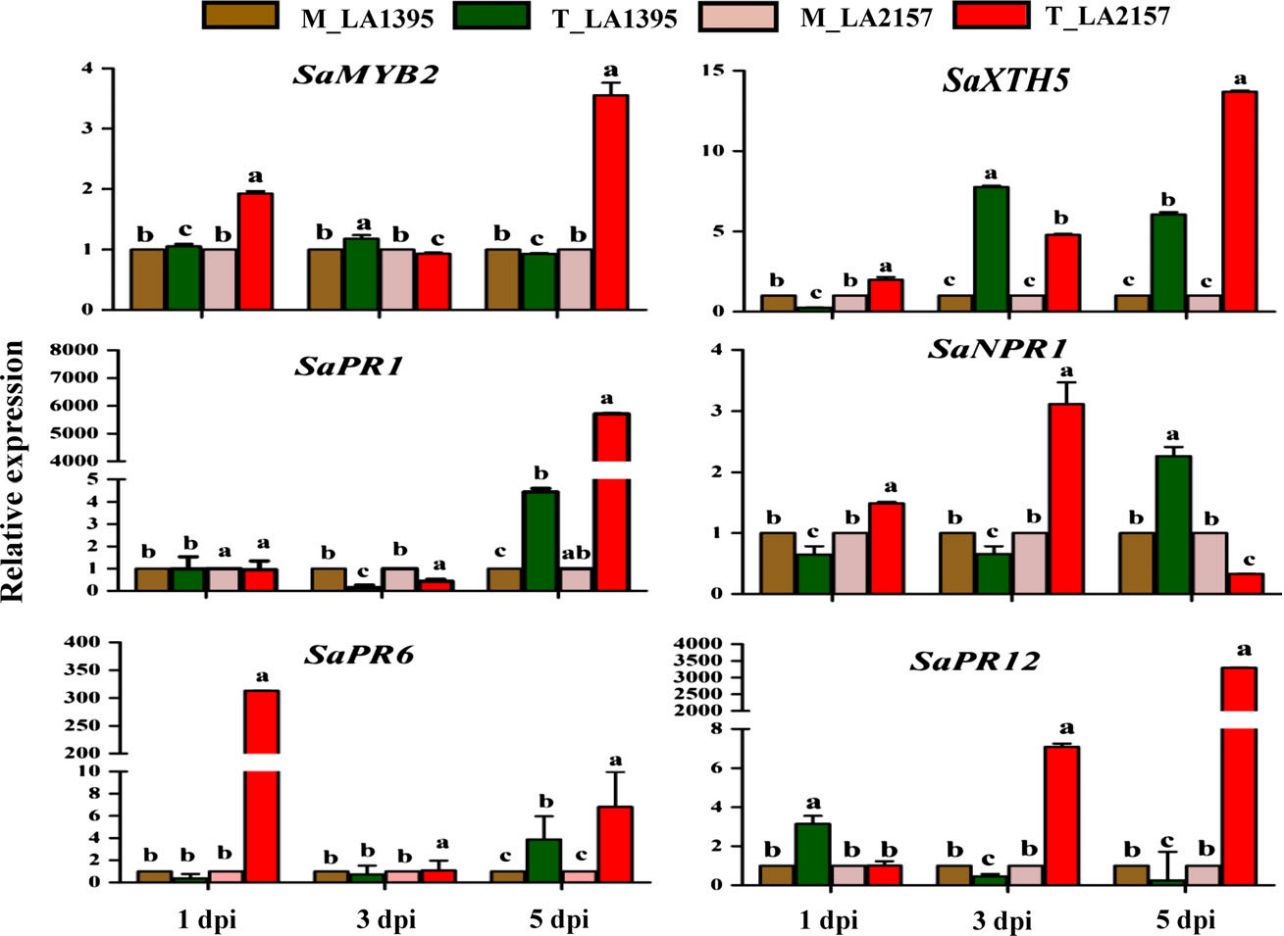
Expression profiles of selected defence genes in susceptible (LA1395) and resistant (LA2157) *Solanum arcanum* accessions. Inoculated with *Alternaria solani* spore suspension (3–4 × 10^3^ spores/mL), leaf samples were collected at 1, 3, and 5 dpi. qRT‐PCR was carried out with *SlEF1α* gene as internal control, and expression was normalized to the corresponding mock (M) and *A. solani* spore‐inoculated (T) samples. The values represent means ± SE of three biological replicates each with three technical replicates. Bars represent the standard errors of the means. Different letters indicate significant differences according to Duncan's test (*P *<* *0.05). Similar pattern of gene expression was obtained in two independent experiments. dpi, days post‐inoculation; M, mock inoculated; T, inoculated with *A. solani* spores; Sa, *S. arcanum* Peralta.

**Figure 4:**
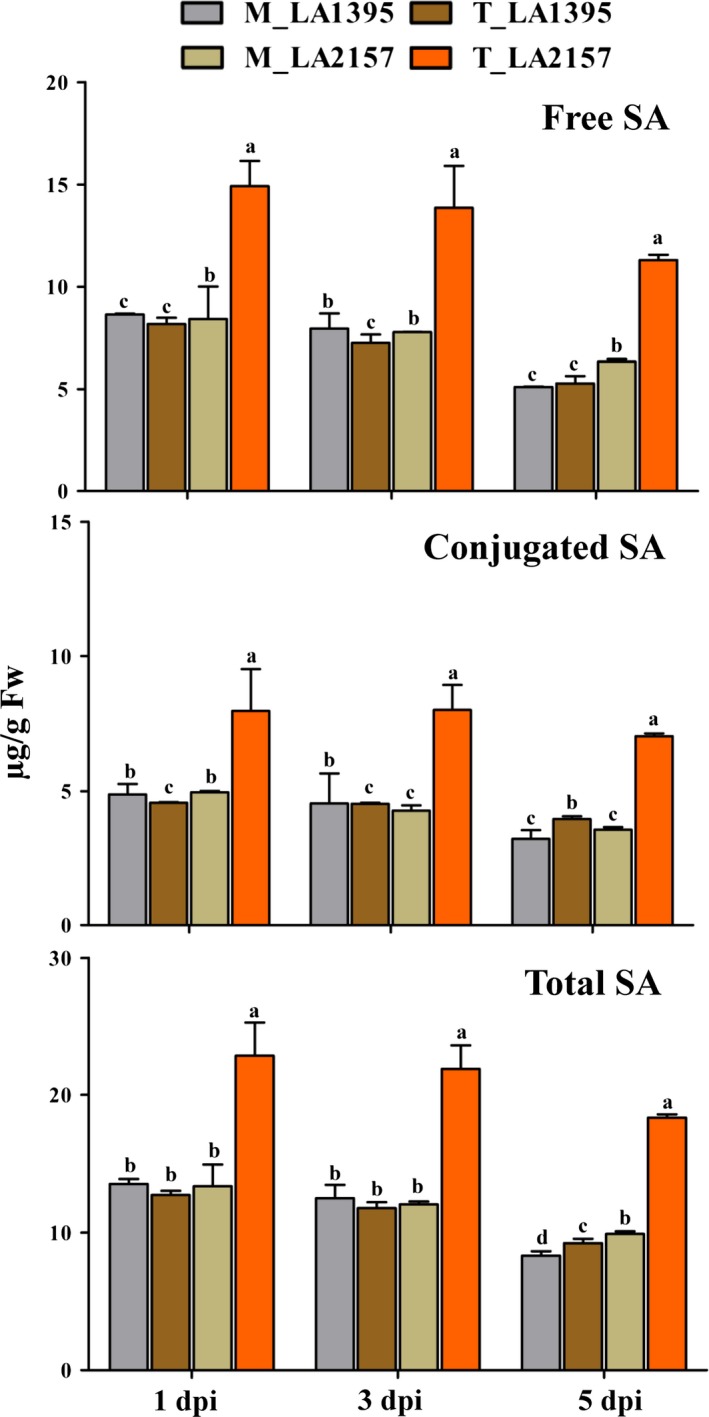
Salicylic acid (SA) content (measured as free, conjugated and total SA μg/g Fw) in mock (M) and *Alternaria solani*‐inoculated (T) leaf tissue of LA2157 (R) and LA1395 (S) *Solanum arcanum* accessions. The values represent means ± SE of three biological replicates each with three technical replicates. Different letters (a, b, c and d) indicate significant differences according to Duncan's test (*P *<* *0.05).

Based on the unique CEG from *WRKY* co‐expression network and promoter analyses, W‐box containing 18 defence genes generated clear differential expression profiles in R and S accessions. Time‐course expression analysis revealed significant increase in transcript levels of *SaMYB2* (up to 3.5‐fold), *SaXTH5* (up to 13.6‐fold)*, SaPR1* (up to 5712‐fold), *SaNPR1* (up to 3.1‐fold), *SaPR6* (up to 313‐fold) and *SaPR12* (up to 3292‐fold) in R compared to S plants upon *A. solani* inoculation (Figure 3). Similarly, most of other defence genes [*SaPOX* (up to 18‐fold), *SaCIPK4* (up to 16.5‐fold), *SaMAPK3* (up to 3.8‐fold), *SaGEBG6* (up to 831.7‐fold), *SaBG* (up to 1213.5‐fold), *SaPR3* acidic (up to 922.9‐fold), *SaPR7* (up to 36.8‐fold)*, SaSTPK* (up to 26.4‐fold)*, SaPR2* (up to 10.5‐fold)*, SaPR3* basic (up to 5.6‐fold) and *SaMYB7* (up to 4.4‐fold)] were significantly up‐regulated in R accession (Figure S2). Further, key biosynthetic genes of SA (*PAL* and *ICS1*) and JA (*AOS* and *OPR3*) depicted contrasting patterns which coincided with stages of EB disease progression (Figure S3). *SaICS1* and *SaPAL* levels were elevated (up to 3‐fold and 6.7‐fold, respectively) in *A. solani*‐treated R compared to S accessions at early stages. However, there was no significant induction in these two genes in both accessions at 5 dpi. Additionally, total SA levels (including free and conjugated forms of SA) remained similar in mock samples of both accessions at different stages. However, these SA contents were significantly high in pathogen‐challenged R plants compared to S (Figure 4) indicating potential involvement of SA in early EB defence. On the other hand, JA biosynthetic genes (*SaAOS* and *SaOPR3*) did not show any elevation in R accession at 1 and 3 dpi but were significantly raised (up to 11.3‐fold and up to 8.9‐fold, respectively) at 5 dpi. Also, *SaJAZ*1 (vital for JA perception) was increased (up to 6.5‐fold) in R plants at 5 dpi (Figure S3). Interestingly after exogenous SA application, the EB disease progression was significantly delayed in both *S. arcanum* accessions which were reflected by lower PDI (>20%–50% decrease in S while >10% in R accession) (Figure S4). These plants also had significant increase in *WRKY1* expression (up to 2.5‐fold) levels upon SA treatment without pathogen infection in both the accessions. This increase was also accompanied by higher levels of *XTH5* and *MYB2* (Figure S5). Expression of these genes, further, rose (up to 14‐fold in *XTH5* and up to 4‐fold in *MYB2*) along with induction in *WRKY1* (up to 4.2‐fold) after pathogen inoculation. Together these findings revealed possible significance of *WRKY1* CEG and SA during early EB defence in wild tomato.

### Nuclear localized SaWRKY1–structurally conserved and closely related to other WRKY1

Of these 12 *WRKY*s, not much is known about the role of *WRKY1* in defence response in wild plants. Interestingly, *WRKY*1 expression was not significantly induced after *A. solani* inoculation in cultivated tomato (*S. lycopersicum*, which lacks EB resistance) and S accession (Figure S6). Further, SA as well as *NPR1* expression levels was increased that coincided with *WRKY1* in R accession at early stages of EB disease. Therefore, we selected *WRKY1* as candidate to obtain its molecular insights during EB defence. On the basis of Arabidopsis WRKY classification and protein sequence analysis, SaWRKY1 and SlWRKY1 proteins had similar N‐ and C‐terminal WRKY domains (NTD and CTD, respectively) with conserved Cys residues which belonged to Group I with zinc‐finger structure of C2H2 type organized in WRKYGQK‐X_13_‐C‐X_4_‐C‐X_22‐23_‐H‐X_1_‐H pattern. SaWRKY1 contained nuclear localizing sequence ‘KRRK’ as predicted by WoLF PSORT tool (Figure S7a). Further, secondary protein structure displayed stronger resemblance between SaWRKY1, SlWRKY1 and available AtWRKY1‐C crystal structure (PDB ID: 2ayd). SaWRKY1 CTD and NTD shared five β‐sheets (β1‐β5) and β‐turns with other two WRKY1 (Figure S7b). To understand the evolutionary relationship of SaWRKY1 with other known 21 plant homologs, NJ‐tree was constructed with their protein sequences. This formed broadly three clusters where SaWRKY1 was closely placed with SlWRKY1 and StWRKY1, while AtWRKY1 was separated in other clusters (Figure S7c). To ascertain cellular localization of SaWRKY1, fusion protein SaWRKY:GFP and only GFP were transiently expressed in tobacco epidermal cells. Fluorescence of GFP was visible in the nuclei from SaWRKY1 fusion protein, confirming its presence in nucleus (Figure S8).

### SaWRKY1 interacted with *SaXTH5* and *SaMYB2* promoters

**Figure 5:**
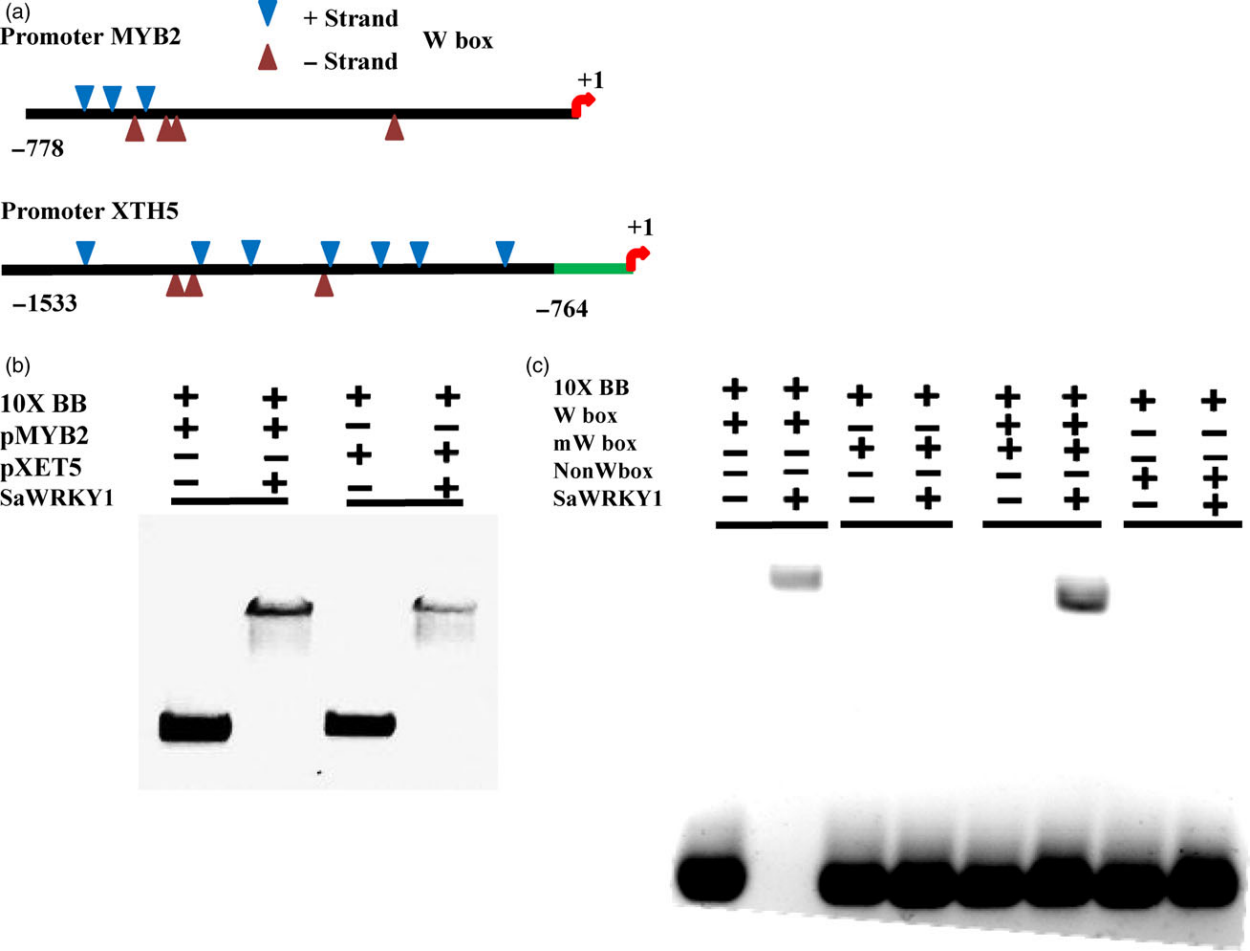
Graphical representation of native *SaMYB2* and *SaXTH5* promoters (a) and interaction of rSaWRKY1 protein with native *SaMYB2* and *SaXTH5* promoters (b) and W‐box probes (c) using EMSA. 10xBB–binding buffer, *pMYB2*–700‐bp native proximal promoter region, *pXTH5*–750‐bp native distal promoter region, rSaWRKY1–recombinant SaWRKY1 protein (62.6 Kda), normal 4xW‐box‐TTGACC, mutated 4xW‐box‐TTtACC, and 4x‐non‐W‐box‐CAATTT as a negative control.

As *SlMYB2* and *SlXTH5* were co‐expressed with *SlWRKY1*, we hypothesized that *SaWRKY1* might physically interact with their promoters and regulate expression of *SaMYB2* and *SaXTH5*. In case of *SaXTH5* promoter, W‐boxes were located at distal promoter region, which included five TTGAC, two TTGACT‐type W‐like boxes and three TGACC‐type W‐like boxes. Similarly in *SaMYB2* promoter, W‐boxes were located at proximal promoter region with two TTGAC, three TTGACT‐type W‐like boxes and two TGACC‐type W‐like boxes (Figure 5a). Further, competitive electro‐mobility shift assay (EMSA) was performed using *SaMYB2* and *SaXTH5* native promoters, 4xW‐box (TTGACC), mutated 4xW‐box (TTtACC) and non‐4xW‐box probes (CAATTT) with rSaWRKY1 protein (Figure S9). Gel shift was clearly evident as rSaWRKY1 was bound to both native promoters and also with W‐box probe (Figure 5b); however, mutated and non‐W‐box probes did not show any gel shift (Figure 5c). This suggested that SaWRKY1 physically bind to *SaXTH5* and *SaMYB2* promoters in sequence‐specific manner.

### Comparative promoter analysis of *WRKY1* portrayed variation in TF‐binding motifs

**Figure 6:**
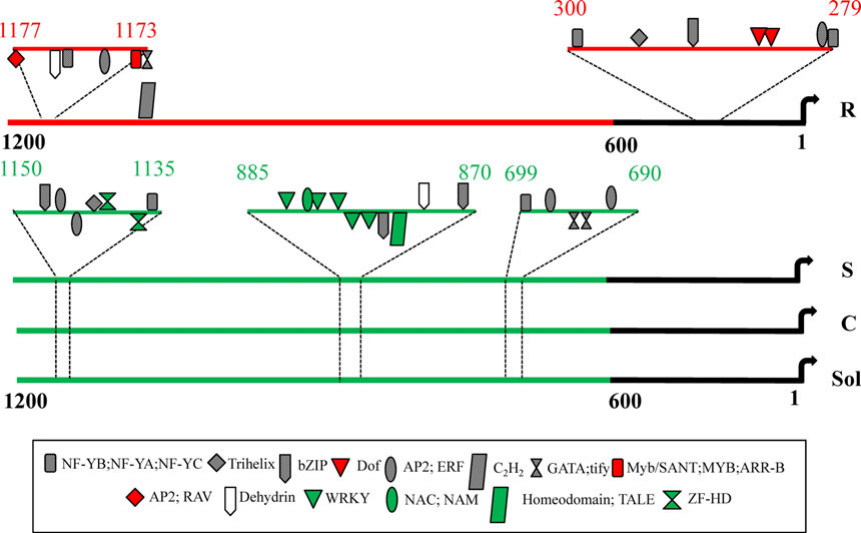
Graphical representation of *WRKY1* (1–1200 bp from TSS) promoter showing binding site of different TFs (as indicated by various shapes and colour marking in the box) from different tomato genotypes. R–LA2157 (resistant) and S–LA1395 (susceptible) *Solanum arcanum* accessions; C–cultivated *S. lycopersicum*; Sol–sequence reported in Sol genomic network webpage, 1–600 bp is considered as proximal, while remaining part as distal region in *WRKY1* promoter.

Further to understand, if nucleotide variations in *WRKY1* promoter have any role in EB phenotypic variation in tomato plants, *WRKY1* promoter regions were cloned from R, S and cultivated tomato plants. After sequencing, these promoter regions were analysed for the variation in TF‐binding sites. Clear differences were observed in the proximal as well as distal *WRKY1* promoter regions in R compared to S and other tomato plants (Figure 6). Unique proximal region of *WRKY1* promoter from R accession harboured *Dof*‐binding motifs which are essential in plant defence while the distal region contained *MYB‐* and *AP2*‐binding regions (Figure 6). On the other hand, these proximal and distal regions were absent in the *WRKY1* promoters from S and cultivated tomato plants. Consequently, other three different distal regions (690–699 bp, 870–885 bp and 1135–1150 bp upstream of TSS) revealed the presence of other TF‐binding sites in S and cultivated tomato (Figure 6). Thus, such variations in TF‐binding motifs in *WRKY1* promoters might have influenced the final outcome of EB phenotype.

### Overexpression and RNAi silencing plants demonstrated positive influence of *WRKY1* in EB defence

Potential contribution of *WRKY1* in EB defence response was further validated using *SlWRKY1* overexpressing (W1OE) in T_1_ generation and silencing (W1RNAi) T_0_ generation tomato plants. As T_0_ W1RNAi silencing transgenic plants had poor seed setting, we were unable to get viable T_1_ generation. As previously shown in cotton, this might be due to dual role of *WRKY1* in plant growth and development as well as in defence (Li *et al*., 2014), and thus, *WRKY1* silencing might have affected seed setting resulting into loss of viable T_1_ generation. Two independent W1OE lines (W1OE‐1 and W1OE‐8) exhibited significant elevation of *SlWRKY1* transcripts (15‐ and 21‐fold, respectively) compared to wild‐type (WT) plants in the absence of pathogen (Figure S10a). *SlMYB2* expression levels also increased significantly in W1OE‐1 and W1OE‐8 (1.7‐ and 1.6‐fold, respectively) compared to WT (Figure S10b). Similarly, these two W1OE lines depicted significant increase in *SlXTH5* expression (>2‐fold) (Figure S10c). On the contrary, five independent W1RNAi (T_0_) lines (W1RNAi‐15, W1RNAi‐21, W1RNAi‐28, W1RNAi‐29 and W1RNAi‐30) had reduced *SlWRKY1* (up to 4‐fold) levels in comparison with WT without pathogen inoculation (Figure S10a). *SlMYB2* and *SlXTH5* indicated significant decrease (up to 5‐fold) in W1RNAi lines as compared to WT (Figure S10b,c).

**Figure 7:**
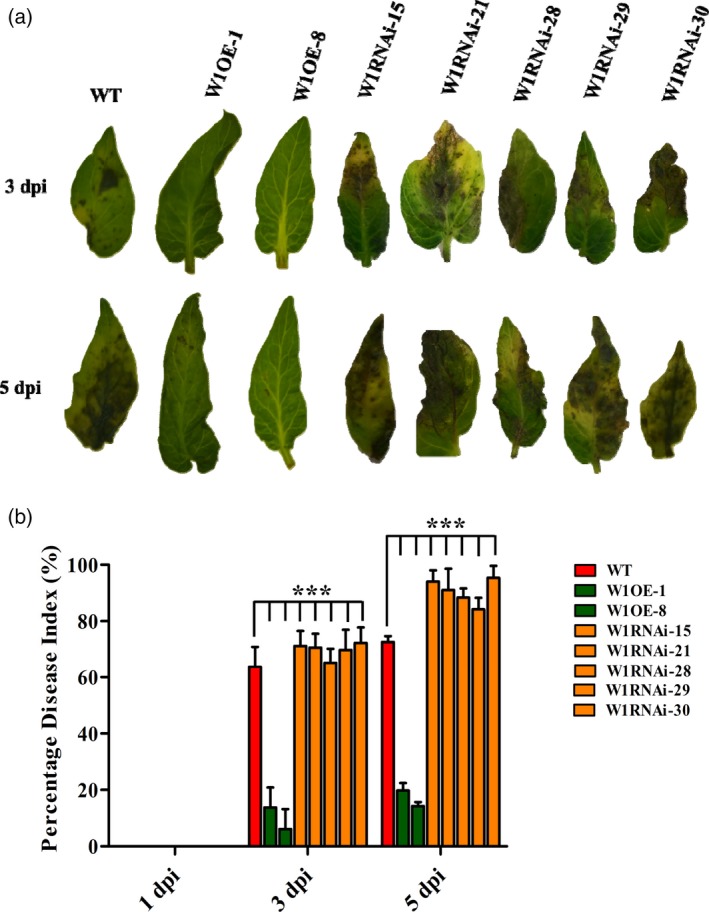
Early blight disease symptoms on leaflets of *SlWRKY1* transgenic lines; a representative leaflet was photographed from each treatment at 3 and 5 dpi (a). Early blight disease scoring in terms of PDI (%) on *SlWRKY1* transgenic tomato lines at 1, 3 and 5 dpi (b). (*SlWRKY1* OE: W1OE‐1, W1OE‐8; *SlWRKY1* RNAi: W1RNAi‐15, W1RNAi‐21, W1RNAi‐28, W1RNAi‐29, W1RNAi‐30, WT: wild‐type nontransformed). The values represent means ± SE. Bars represent the standard errors of the means. In statistical analysis, two‐way ANOVA was performed followed by Bonferroni post‐tests. Statistical data are significant at *P*‐value ***P <* *0.001.

Upon *A. solani* inoculation, phenotypic assessment of EB symptoms displayed improved resistance with decreased necrotic lesions on leaflets of W1OE‐1 and W1OE‐8 lines (T_1_) in comparison with WT (Figure 7a). On the other hand, all five W1RNAi lines showed increased susceptibility to EB (Figure 7a). Consequently, disease severity (PDI) was also significantly decreased (>50%) in both W1OE lines compared to WT at 3 and 5 dpi. In the case of W1RNAi lines, PDI was elevated significantly (20%–40%) than WT plants (Figure 7b). W1OE lines exhibited significant up‐regulation of *WRKY1, MYB2* and *XTH*5 (up to 20‐, 2.5‐ and 45.9‐fold, respectively) whereas in case of W1RNAi lines, these were down‐regulated (up to 10‐fold) upon pathogen challenge (Figure S11). Moreover, expression of *SlPAL*,* SlICS1* and *SlPR1* was also significantly elevated (up to 7.2, 118.5 and >150‐fold, respectively) in W1OE lines upon *A. solani* inoculation while reduction (up to 3‐fold) was observed in W1RNAi lines (Figure S12). Similarly, *SlAOS*,* SlOPR3*,* SlPR12* and *SlJAZ1* levels were significantly high (up to 130.6‐, 5.7‐, 8.8‐ and >150‐fold, respectively) in these W1OE lines compared to WT (Figure S13). Contrary, expression of *SlOPR3*,* SlPR12* and *SlJAZ1* was reduced (up to 2‐fold) at 3 dpi but showed elevation (up to 1.86‐fold) at 5 dpi upon *A. solani* inoculation (Figure S13). Taken together, transgenic plants demonstrated critical involvement of *WRKY1* during EB defence**.**

## Discussion

Wild relatives of crops represent potential gene pool and thus, are primary source of important genes (Foolad, 2007). Phenotypic comparison of LA2157 and LA1395 accessions of *S. arcanum* in EB disease severity indicated that LA2157 has robust resistance against *A. solani*. QTL analysis of *S. lycopersicum* cv. ‘Solentos’ x *S. arcanum* (LA2157) population has identified six QTLs governing resistance to EB (Chaerani *et al*., 2007). Recently, high‐throughput metabolomic study revealed positive effect of secondary metabolites from steroidal‐glyco alkaloid and phenylpropanoid pathways in protecting wild tomato against EB (Shinde *et al*., 2017). However, there are no reports available until now describing molecular mechanism(s) of EB resistance in wild relatives or cultivated plants. Current investigation suggested significant modulation in 12 *WRKY* transcripts at early and late stages of EB disease progression and majority of these were significantly up‐regulated in R accession indicating their potential involvement in resistance against *A. solani*. In Arabidopsis, *AtWRKY8* expression was increased with pathogen infection, aphid and maggot feeding while loss of function mutants showed reduction in resistance to *Botrytis cinerea* (Chen *et al*., 2010). Similarly, *AtWRKY19* displayed recessive resistance to several strains of *Ralstonia solanacearum* (Deslandes *et al*., 2003). Also *PtWRKY23* transcripts were elevated in Populus after *Melampsora medusa* infection and elicitor treatments (Levée *et al*., 2009) and *AtWRKY23* provided resistance against nematode infection (Grunewald *et al*., 2008). *AtWRKY*22 was up‐regulated against bacterial and fungal pathogens (Dong *et al*., 2003); however, it induced susceptibility to aphid (Kloth *et al*., 2016).

Earlier, *WRKY1* and *WRKY33* have been shown as SA responsive markers for early plant defence while *WRKY33* and *WRKY40* as late stage‐specific response (Bakshi and Oelmuller, 2014). Nonetheless, present findings indicated that all these three *WRKYs* together with *WRKY11* were potentially involved in EB resistance. SA treatment has resulted into *AtWRKY1* induced expression which is dependent on *NPR1* (Duan *et al*., 2007) while SA‐independent expression of *WRKY33* is known to be essential in necrotrophic defence (Bakshi and Oelmuller, 2014). Elevated *AtWRKY33* expression was reported in response to *B. cineria* (Zheng *et al*., 2006). During BABA‐induced resistance, necrotrophic inoculation resulted in higher levels of *BjWRKY11* and *SlWRKY11* in brassica and tomato, respectively (Chavan and Kamble, 2013; Roylawar *et al*., 2015). Consistence with above reports, *SaWRKY11* and *SaWRKY40* levels were increased in R accession. Additionally, key genes of SA and JA biosynthesis (*PAL*,* ICS1*,* AOS* and *OPR3*) as well as their defence markers (*PR1*,* PR2*,* PR6* and *PR12*) were also up‐regulated in R accession. *ICS1* regulates important step in SA biosynthesis and shown to be regulated by *WRKYs* (VanVerk *et al*., 2011). Similarly, *PAL* isoforms play critical role in SA biosynthesis and lignification (Gayoso *et al*., 2010) and increased lignin accumulation was observed in resistant compared to susceptible plants upon *A. solani* inoculation (Shinde *et al*., 2017). Overexpression of *NtPAL* resulted in the increased resistance to TMV and *Cercospora nicotianae* (Shadle *et al*., 2003) while silencing of *WRKY53* has resulted in lower *PAL* expression with increased susceptibility to aphids in wheat (Van Eck *et al*., 2010). Therefore, SA could be involved at early stage of EB resistance in R based on the significant increase in *SaICS1* and *SaPAL* transcripts. Pathogen‐treated R accessions clearly displayed significantly high SA levels compared to S plants. Exogenous SA application significantly delayed the EB disease progression as well as increased *WRKY1* expression in S and R accessions at early stage upon *A. solani* challenge. WRKY1 governs SA signalling pathway through cytosolic form of NPR1 that acts as a critical master regulator of plant defence response and is tightly governed through post‐translation modifications (Saleh *et al*., 2015). In Arabidopsis, mutation in W‐box of *NPR1* promoter completely disrupted binding of WRKYs and abolished *NPR1* expression, resulting in susceptibility against *Pseudomonas syringae* (Yu *et al*., 2001). Similarly, Arabidopsis *npr1* mutants upon *P. syringae* infection failed to trigger *PR* genes and were susceptible to a wide range of pathogens (Conrath *et al*., 2006). Heterologous overexpression of *AtNPR1* showed resistance to *Xanthomonas oryzae* pv. *oryzae* in rice (Chern *et al*., 2001). Thus, significant *SaNPR1* elevation in R might suggest its role in early resistance against *A. solani*. Moreover, SA‐defective mutants (*pad4* and *sid2*) revealed more resistance at early stages against *A. brassicicola* in Arabidopsis (VanWees *et al*., 2003). Taken together, a minimum threshold level of SA along with *WRKY1* and other defence genes at initial stage might be critical for EB defence.

Comparison between CTD and NTD of SaWRKY1 with secondary structure of AtWRKY1‐C revealed similar functional domains with important conserved binding sites. Structural and phylogenetic studies revealed that SaWRKY1 was closely related to its homologs in tomato and potato (Agarwal *et al*., 2011). Comparative *WRKY1* promoter analysis clearly depicted that variation in the TF‐binding regions (Wen *et al*., 2016) could be responsible for the differential *WRKY1* expression in R and other susceptible tomato cultivars and thus, might improve defence potential of resistance accession against EB. Furthermore, promoter analysis and EMSA clearly provided critical evidences for *SaXTH5* and *SaMYB2* as the targets of *SaWRKY1*. XTH plays important role in cell wall modulation (Hayashi and Kaida, 2011; Nishikubo *et al*., 2011), and *LeXTH* was up‐regulated during incompatible tomato‐*Cuscuta* interaction indicating its importance in plant defence (Albert *et al*., 2004). Similarly, *MYB2* has been implicated in hormonal crosstalk during plant defence response (VanVerk *et al*., 2011). Transcript levels of *SaXTH5* and *SaMYB2* were significantly up‐regulated in R accession upon *A. solani* inoculation and might unveil their contribution in restricting this necrotrophic pathogen. Moreover, functional relationships between WRKY1 and its targets (*SlXTH5* and *SlMYB2*) were confirmed in *SlWRKY1* transgenic tomato lines. Both W1OE T_1_ lines displayed improved EB resistance while five independent W1RNAi T_0_ lines exhibited higher EB susceptibility. However, T_1_ W1RNAi lines in this study failed due to poor seed setting and experimental validation of this observation (whether it is due to lethality or male sterility) may require a separate study. Interestingly, overexpression of *GbWRKY1* has indicated multiple roles including organ development and fungal resistance in cotton (Li *et al*., 2014). Heterologous overexpression of *VvWRKY1* in tobacco exhibited reduced susceptibility to several fungi (Marchive *et al*., 2007). Transgenic disease‐resistant rice has been developed against *Magnaporthe oryzae* and *X. oryzae pv. oryzae* by expressing *WRKY45* under *PR* promoter (Goto *et al*., 2016). Similarly, *SlDRW1* has been found to regulate defence response against *B. cinerea* in tomato (Liu *et al*., 2014). Overall, significant variation in TF‐binding motifs in *WRKY1* promoter regions and differential *XTH5* and *MYB2* expression could be crucial during EB defence. Transgenic tomato lines indicated that *WRKY1* might have a vital role in resistance against *A. solani*. Current findings offer critical evidences for the role of *WRKY1* in modulation of EB resistance in tomato and could be potentially utilized to improve plant defence against *A. solani* in other crops.

## Experimental procedures

### Co‐expression network analysis of *WRKYs* and gene ontology prediction of CEG

Co‐expression analysis was performed on available transcriptomic data (Itkin *et al*., 2011, 2013) using R script, and this generated a separate list of CEG for each group with *r *≤* *0.8 and sorted in descending order. Co‐expression networks were visualized with the Cytoscape program version 3.1.0 (Shannon *et al*., 2003). GO annotation of CEG was performed according to Barvkar *et al*. (2013) with plant‐specific database and expression value ≤e^−20^ (McCarthy *et al*., 2006).

### 5′ *cis*‐regulatory element analysis

For each gene with defence‐related GO annotation (for categories like response to stimulus, immune system process and biological regulation), ~1.5 Kb of 5′ regulatory region from the translational start site was retrieved from Sol genomic network (https://solgenomics.net) associated search tools (Fernandez‐Pozo *et al*., 2015). Presence of putative 5′ *cis*‐regulatory elements was detected in these promoters using PlantPAN database 2.0 (Chow *et al*., 2015).

### Plant material, fungal culture, EB disease assessment and SA application

LA2157 (R) and LA1395 (S) accessions of *S. arcanum* were procured from Tomato Genomic Research Centre, University of California, Davis, USA, while seeds of cultivated tomato (*S. lycopersicum* cv. Naina) were acquired from local market. In case of exogenous SA application, 1 mM solution of SA was spread on whole plant till run off on the 1‐month‐old R and S plants. Conditions of plant growth, *A. solani* culture and EB disease phenotyping were as per Pandey *et al*. (2003) and Shinde *et al*. (2017).

### Sample collection, RNA isolation and qRT‐PCR analysis

Leaf samples from mock‐ and pathogen‐inoculated, normal (R and S), wild‐type and transgenic and SA‐treated (R and S) plants were simultaneously collected for respective experiments, and RNA was isolated from all samples. For qRT‐PCR, Gene Runner software was utilized to develop all gene‐specific primers (Table S7). The phenotypic data and gene expression results are presented as mean ± SE of three independent biological with three technical replicates of each (Schmittgen and Livak, 2008; Shinde *et al*., 2017).

### Cloning, secondary protein structure and phylogenetic analyses of SaWRKY1

Full‐length *SaWRKY1* sequence was amplified from cDNA of LA2157, cloned in pCloneJET vector (Fermentas, Waltham, MA) and sequenced. Secondary structure of SaWRKY1, CTD and NTD, was predicted based on known crystal structure of Arabidopsis WRKY1‐C (PDB ID: 2ayd) using ESPript (http://espript.ibcp.fr). Phylogenetic analysis was performed in MEGA6 (Tamura *et al*., 2013) using the Neighbour‐Joining (NJ) method with 1000 iterations using SaWRKY1 along with other 21 homologs.

### Nuclear localization of SaWRKY1

Coding sequence (CDS) of *SaWRKY1* and GFP was amplified (Table S7). Both *SaWRKY1* and GFP were cloned in binary plant expression pRI101‐ANvector (Takara Bio Inc., Kusatsu, Shiga, Japan). *pRI101‐AN:SaWRKY:GFP* and *pRI101‐AN:GFP* clones were transformed into *Agrobacterium tumefaciens* strain GV3101 using a standard transformation protocol. Empty *pRI101‐AN* vector was used as a negative control. *A. tumefaciens* cultures with respective clones were grown at 28 °C in Luria‐Bertani medium containing selection markers (25 mg/L rifampicin and 50 mg/L kanamycin) at 120 rpm for 24 h. Cells were harvested by centrifugation at 1370 ***g*** for 5 min and suspended in infiltration buffer (half strength MS medium, pH 5.6 and 200 μM acetosyringone). These were pelleted and re‐suspended in infiltration buffer by adjusting an OD 1.0 at 600 nm. Cultures were incubated at 24 °C for 3–4 h and were infiltrated into leaves of 2‐week‐old *Nicotiana benthamiana* plants that were maintained at 18 to 24 °C in a growth chamber. Leaf sections were visualized for subcellular localization of GFP at 6 dpi after agro‐infiltration using confocal laser scanning microscope (Zeiss LSM 710, Oberkochen, Germany).

### rSaWRKY1 expression, purification and EMSA

CDS of *SaWRKY1* was cloned using expression vector–pET32a (Novagen, Darmstadt, Germany); and recombinant protein expressed and purified as per the protocol of Kim *et al*. (2006). *SaXTH5 and SaMYB2* promoters (1.5 Kb upstream of TSS) were cloned in pCloneJET vector (Fermentas) and sequenced (Eurofin, Bengaluru, India). For EMSA, probes with 4xW‐box, 4x‐non‐W‐boxes and 4x‐mutated‐W‐box were designed (Table S7). Sense and antisense above‐mentioned probes with equi‐molar concentration were annealed using binding buffer (containing 0.10 mg/mL BSA, 1 mM DTT, 10 mM EDTA, 50% v/v glycerol, 1 M KCl and 100 mM Tris‐HCl pH 7.5) by heating (95 °C for 15–20 min) and then cooling the mixture at room temperature. rSaWRKY1 protein (10 μg) was utilized in EMSA with *pXTH5* and *pMYB2* promoters (100 ng) and with 4xW‐box probes. Respective reaction mix was incubated at 25 °C for 30 min. Separation and visualization of DNA–protein complexes were carried out using 2% agarose gel electrophoresis and staining with 0.05% GelRed (Biotium, Fremont, CA), respectively.

### Cloning and promoter analysis of *WRKY1*

Promoter regions of *WRKY1* (1.2 Kb, upstream of TSS) from resistant (LA2157), susceptible (LA1395) *S. arcanum* accessions and cultivated *S. lycopersicum* were cloned in pCloneJET vector (Fermentas) and were sequenced (Eurofin). The presence of putative 5′ *cis*‐regulatory elements was detected in these promoters using PlantPAN database 2.0 (Chow *et al*., 2015).

### Generation of *WRKY1* overexpression and RNAi silencing transgenic plants

For construction of *SlWRKY1* overexpression (W1OE) and silencing (W1RNAi) vectors, full‐length CDS of *SlWRKY1* cloned in pDONR221 vector was transferred in pK2GW7 binary vector and its 265‐bp fragment cloned in pENTR/D‐TOPO was transferred in pK7GWIWG2 (II) binary vector, respectively, according to GATEWAY technology with respective clonase II enzyme mix (Invitrogen, Carlsbad, CA) (Cárdenas *et al*., 2016). Further, constructs were transformed into *S. lycopersicum* cv Microtom as background using modified protocol of Meissner *et al*. (1997) to generate *SlWRKY1* overexpression and RNAi silenced lines. Expression levels of *SlWRKY1, SlXTH5* and *SlMYB2* were used to screen the W1OE (T_0_, four independent transgenic plants) and W1RNAi (T_0_, eight independent transgenic plants). Based on this, two each independent W1OE (T_1_ transgenic generation) and five each independent W1RNAi (T_0_ transgenic generation) transgenic plants were used in further study. EB disease assessment [by detached leaf method (http://agris.fao.org/aos/records/US201400113953)] as well as expression levels of *SlWRKY1, SlXTH5* and *SlMYB2* along with key genes was performed on T_1_ W1OE lines, T_0_ W1RNAi lines and their respective WT plants.

### SA estimation

Ground leaf tissue (300 mg) from each collected sample was used for total SA content measurements along with free and conjugated SA according to Schenk *et al*. (2014). SA samples were separated on LiChrospher 100 RP‐18 (5 μm) (Hibar® RT 250‐4 HPLC column; Merck KGaA, Darmstadt, Germany) reverse phase column (25 cm × 4 mm i.d.) at a flow rate of 0.8 mL/min in a HPLC Agilent technologies 1260 infinity series (Santa Clara, CA) equipped with G1321C 1260FLD detector using a gradient programme for the solvent (Eggert *et al*., 2010). Detection of SA was performed using a fluorescence detector (excitation wavelength 315 nm and emission wavelength 405 nm). An injection volume of 20 μL was used in HPLC through autosampler G1329B (1260ALS) and quantified with diluted SA standard (range 20–640 ng/μL in 80% aqueous methanol).

### GenBank accession numbers

SaWRKY1 (KU674828), SaWRKY3 (KU674829), SaWRKY8 (KU674830), SaWRKY11 (KU674831) and SaWRKY40 (KU674832).

## Supporting information


**Figure S1** Gene ontology (GO) analysis of all CEGs.
**Figure S2** Expression profiles of selected defence genes.
**Figure S3** Expression profiles of key SA and JA biosynthetic genes.
**Figure S4** Early Blight disease scoring of R and S plants after exogenous SA application.
**Figure S5** Expression analysis of *SlWRKY1*,* SlXTH5* and *SlMYB2* after exogenous SA application.
**Figure S6** Expression profiles of *WRKY1* in *Solanum lycopersicum* (Sl), susceptible and resistant *Solanum arcanum* accessions
**Figure S7** (a) Protein sequence alignment of SaWRKY1 and SlWRKY1; (b) structure based sequence alignment of SaWRKY1 C and N terminal domains with tomato and Arabidopsis WRKY1; and (c) phylogenetic analysis of WRKY1 using amino acid sequences from 21 plant homologs.
**Figure S8** Subcellular localization of GFP in absence and presence of SaWRKY1.
**Figure S9** SDS‐PAGE of recombinant SaWRKY1 protein.
**Figure S10** Expression analysis of *SlWRKY1*,* SlXTH5* and *SlMYB2* in T_0_ transgenic tomato lines.
**Figure S11** Expression analysis of *SlWRKY1*,* SlXTH5* and *SlMYB2* using qRT‐PCR in transgenic tomato W1OE and W1RNAi lines.
**Figure S12** Expression analysis of key genes involved in SA mediated defence response (*SlPAL*,* SlICS1* and *SlPR1*) using qRT‐PCR in transgenic tomato W1OE and W1RNAi lines.
**Figure S13** Expression analysis of key genes involved in JA mediated defence response (*SlAOS*,* SlOPR3*,* SlJAZ* and *SlPR12*) using qRT‐PCR in transgenic tomato W1OE and W1RNAi lines.

**Table S1** List of co‐expressed genes with five WRKYs (WRKY1, 3, 8, 23, and 39).

**Table S2** List of co‐expressed genes with three WRKYs (WRKY11, 33, and 40).

**Table S3** List of co‐expressed genes with two WRKYs (WRKY4, and 54).

**Table S4** Defence gene with GO terms–Biological regulation, Immune system process and response to stimulus.

**Table S5** Potential 5′ *cis*‐acting elements from identified defence‐related genes and their predicted functions.

**Table S6** Frequency of 5′ *cis* elements in all defence‐related *WRKY* co‐expressed genes.

**Table S7** List of primers used in present study.
