# Supplementary material for: WRKY1 acts as a key component improving resistance against Alternaria solani in wild tomato, Solanum arcanum Peralta
Source: Plant Biotechnol J. 2018 May 24;16(8):1502–13. doi: 10.1111/pbi.12892 (PMC6041441; doi:10.1111/pbi.12892)
Supplement: Supplementary file 1 — Figure S1 Gene ontology (GO) analysis of all CEGs. Figure S2 Expression profiles of selected defence genes. Figure S3 Expression profiles of key SA and JA biosynthetic genes. Figure S4 Early Blight disease scoring of R and S plants after exogenous SA application. Figure S5 Expression analysis of SlWRKY1, SlXTH5 and SlMYB2 after exogenous SA application. Figure S6 Expression profiles of WRKY1 in Solanum lycopersicum (Sl), susceptible and resistant Solanum arcanum accessions Figure S7 (a) Protein sequence alignment of SaWRKY1 and SlWRKY1; (b) structure based sequence alignment of SaWRKY1 C and N terminal domains with tomato and Arabidopsis WRKY1; and (c) phylogenetic analysis of WRKY1 using amino acid sequences from 21 plant homologs. Figure S8 Subcellular localization of GFP in absence and presence of SaWRKY1. Figure S9 SDS‐PAGE of recombinant SaWRKY1 protein. Figure S10 Expression analysis of SlWRKY1, SlXTH5 and SlMYB2 in T0 transgenic tomato lines. Figure S11 Expression analysis of SlWRKY1, SlXTH5 and SlMYB2 using qRT‐PCR in transgenic tomato W1OE and W1RNAi lines. Figure S12 Expression analysis of key genes involved in SA mediated defence response (SlPAL, SlICS1 and SlPR1) using qRT‐PCR in transgenic tomato W1OE and W1RNAi lines. Figure S13 Expression analysis of key genes involved in JA mediated defence response (SlAOS, SlOPR3, SlJAZ and SlPR12) using qRT‐PCR in transgenic tomato W1OE and W1RNAi lines. [file PBI-16-1502-s004.pdf]

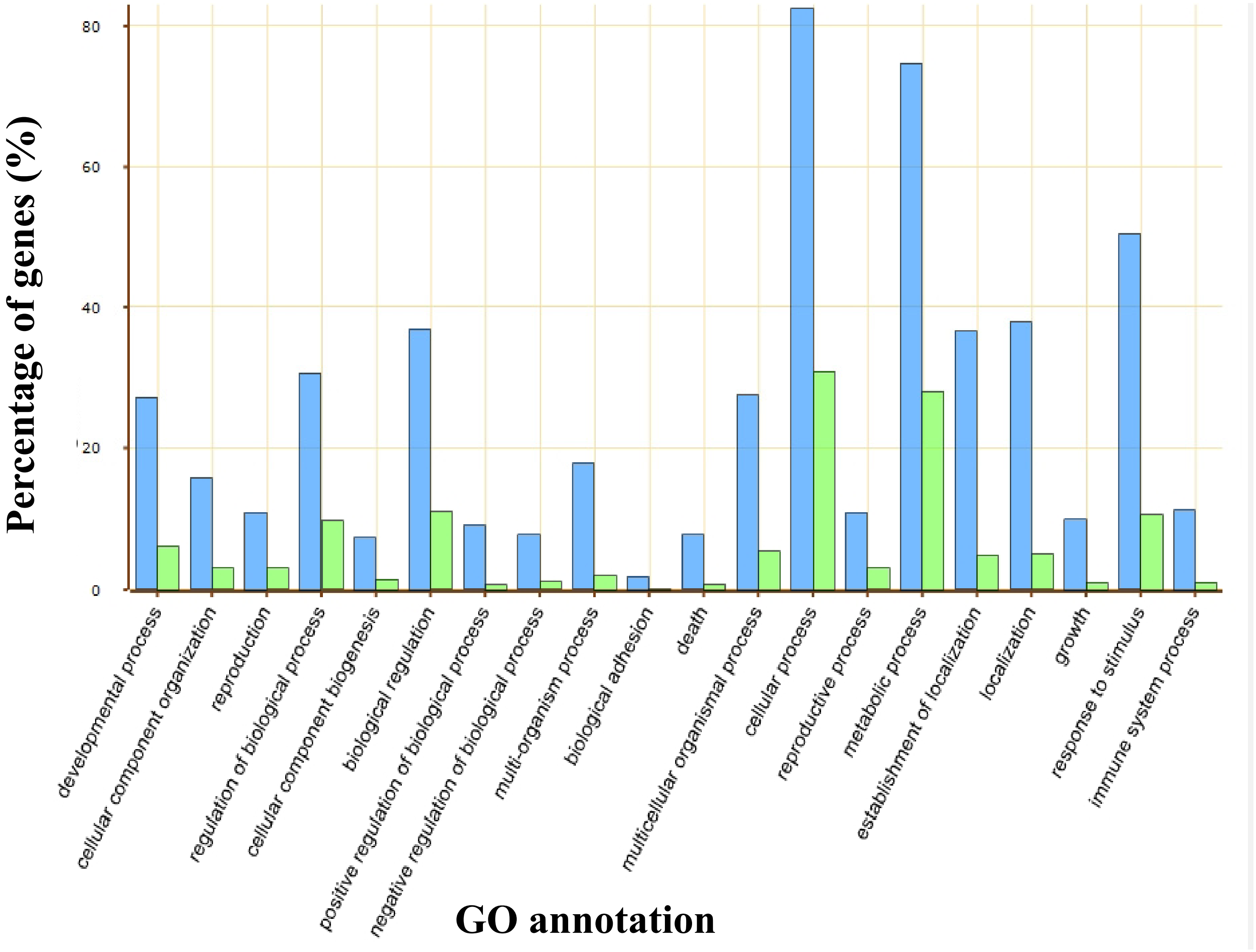

Figure S1. Gene ontology (GO) analysis of all CEGs. Blue bars indicate the enrichment of WRKY CEGs in GO terms. Green bars indicates the percentage of total annotated Arabidopsis genes mapping to GO terms.

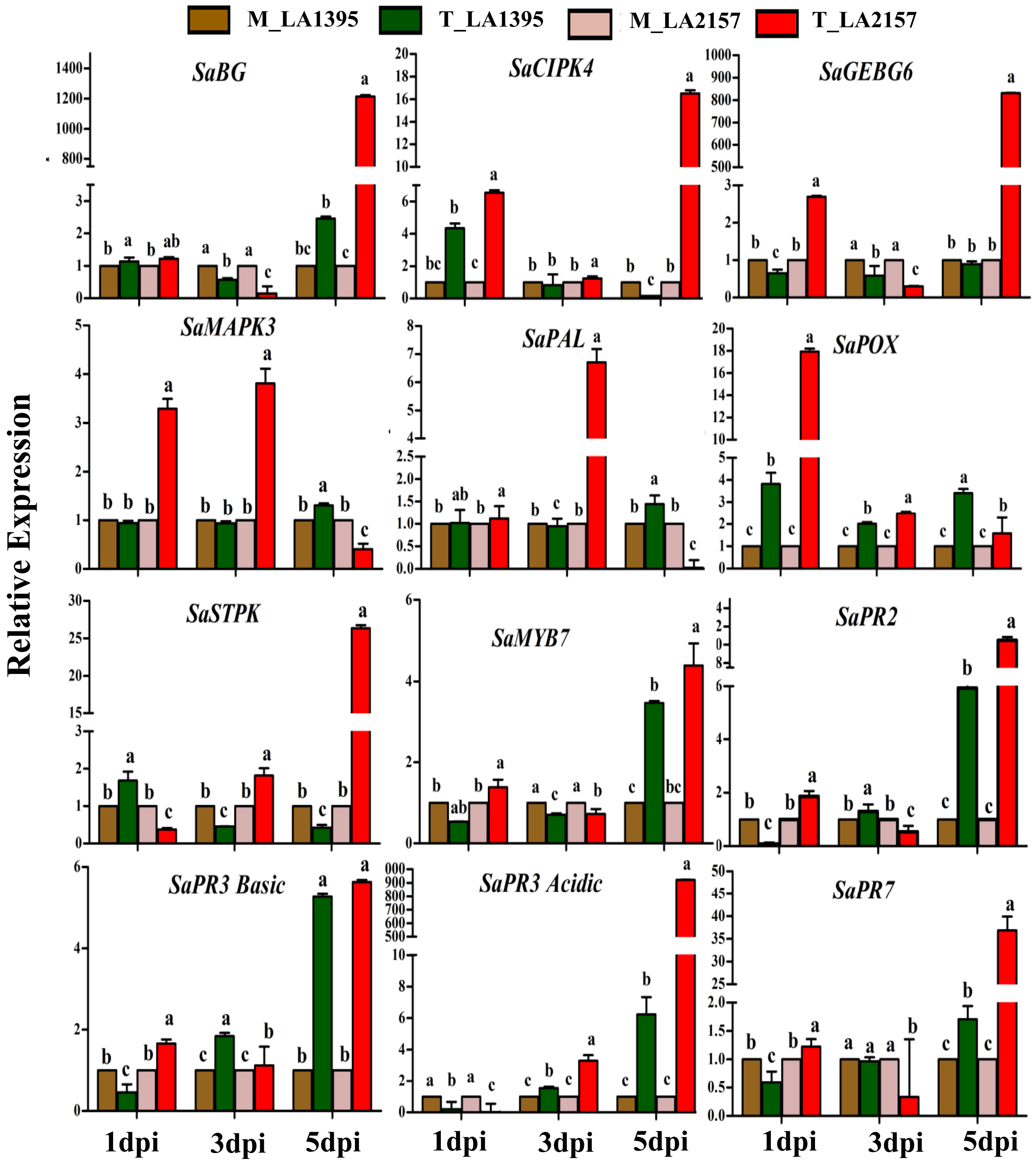

Figure S2: Expression profiles of selected defence genes in susceptible (LA1395) and resistant (LA2157) *S. arcanum* accessions. Inoculated with *A. solani* spore suspension (3-4x10^3 spores/mL), leaf samples were collected at 1, 3, and 5 dpi. qRT-PCR was carried out with *SlEF1α*gene as internal control and expression was normalized to the corresponding *A. solani* spore inoculated (T) and mock inoculated (M) samples. The values represent means ± SE of three biological replicates each with three technical replicates. Bars represent the standard errors of the means. Different letters indicate significant differences according to Duncan's test (P<0.05). Similar pattern of gene expression was obtained in two independent experiments. dpi-days post inoculation, M-Mock inoculated, T- inoculated with *A. solani* spores, Sa- *Solanum arcanum* Peralta.

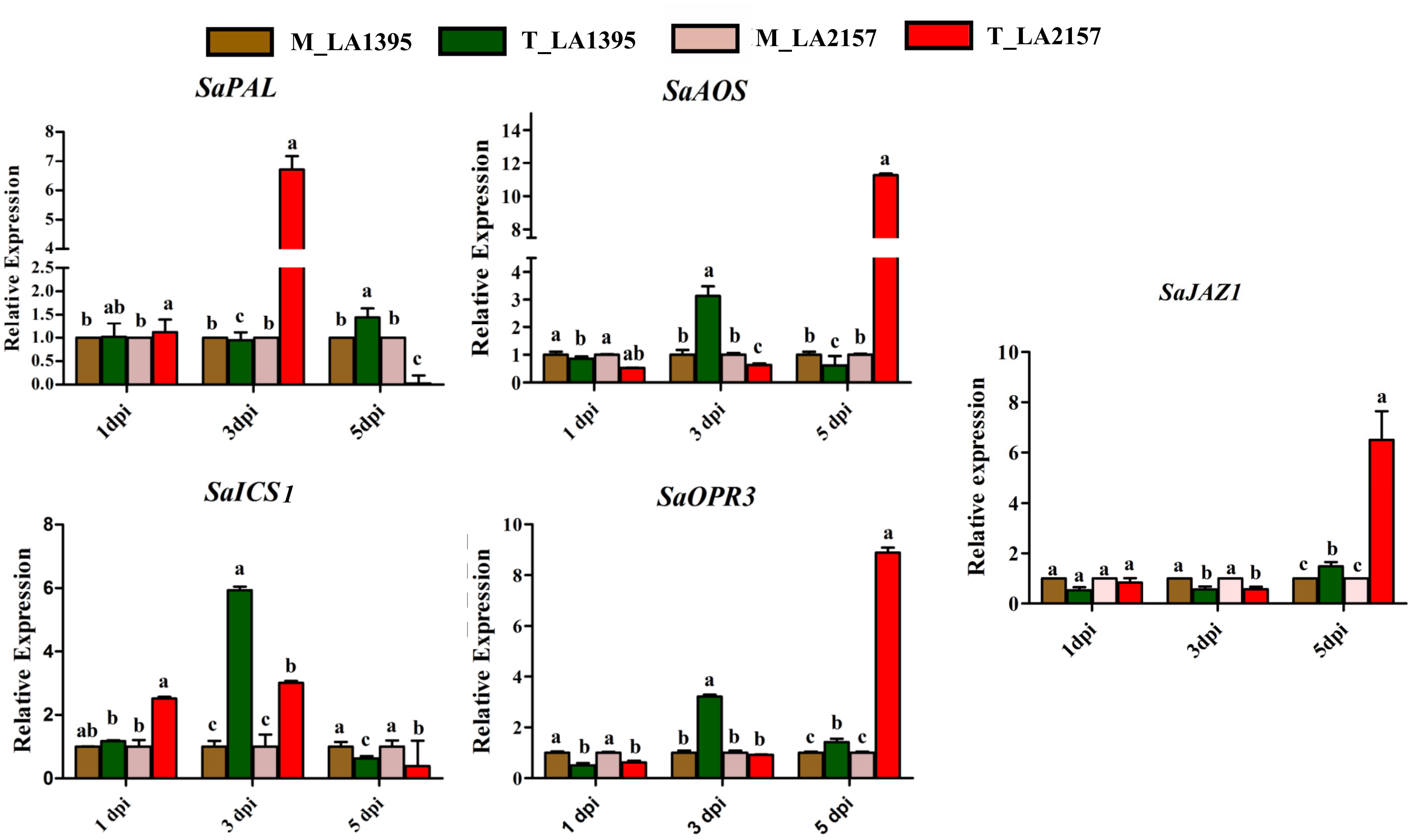

Figure S3. Expression profiles of key SA and JA biosynthetic genes in susceptible (LA1395) and resistant (LA2157) *S. arcanum* accessions. Inoculated with *A. solani* spore suspension (3-4x10^3 spores/mL), leaf samples were collected at 1, 3, and 5 dpi. qRT-PCR was carried out with *SlEF1α* gene as internal control and expression was normalized to the corresponding *A. solani* spore inoculated (T) and mock inoculated (M) samples. The values represent means ± SE of three biological replicates each with three technical replicates. Bars represent the standard errors of the means. Different letters indicate significant differences according to Duncan's test (P <0.05). Similar pattern of gene expression was obtained in two independent experiments. dpi- Days Post Inoculation, M-Mock inoculated, T- inoculated with *A. solani* spores, Sa- *Solanum arcanum* Peralta.

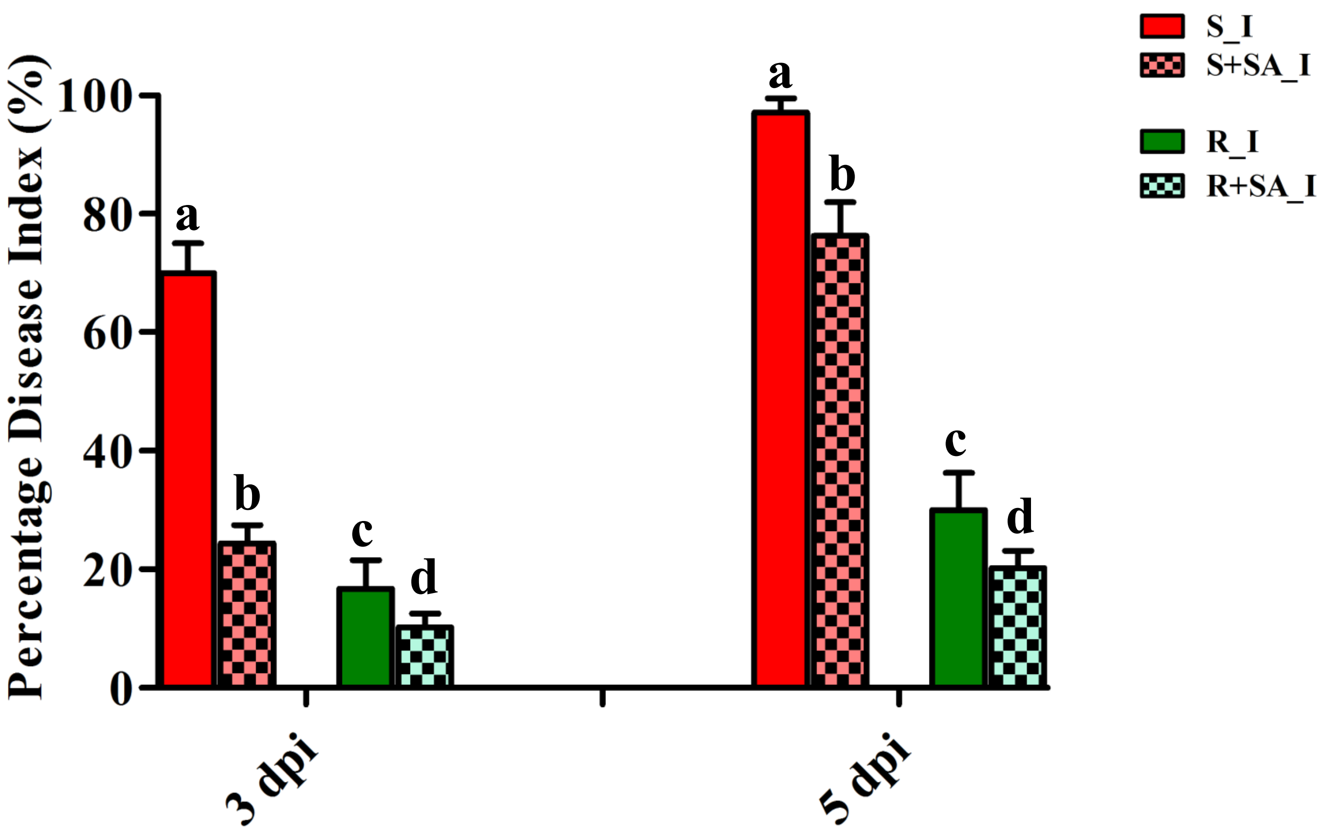

Figure S4. Early blight disease scoring of R and S plants after exogenous application of 1 mM SA at 3 and 5 dpi. S_I: Susceptible *A. solani* inoculated, S+SA_I: Susceptible *A. solani* inoculated after SA treatment R_I: Resistant *A. solani* inoculated, R+SA_I: Resistant *A. solani* inoculated after SA treatment The values represent means ± SE. Bars represent the standard errors of the means. In statistical analysis, Different letters indicate significant differences according to Duncan's test ($P < 0.05$).

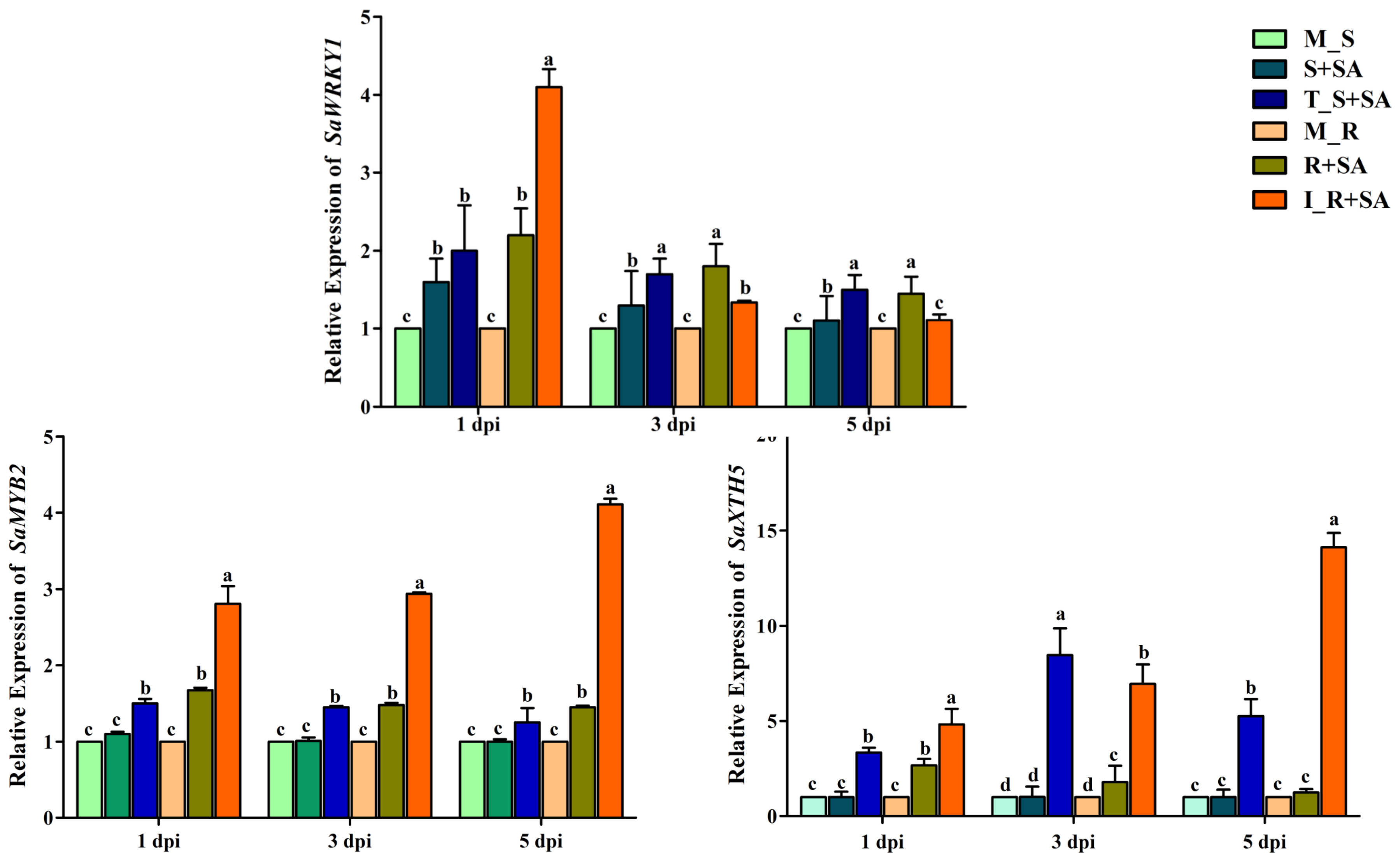

Figure S5. Expression analysis of *SlWRKY1, SlXTH5* and *SlMYB2* using qRT-PCR in R and S plants at 1,3, and 5 dpi after 1mM exogenous SA application qRT-PCR was carried out with *SlEF1α* gene as internal control and expression was normalized to the corresponding *A. solani* spore (T) and mock inoculated (M) samples. Expression variation represents fold change against expression in mock treated WT plants. The values represent means ± SE of three biological replicates each with three technical replicates. Bars represent the standard errors of the means. Different letters indicate significant differences according to Duncan's test (P <0.05).

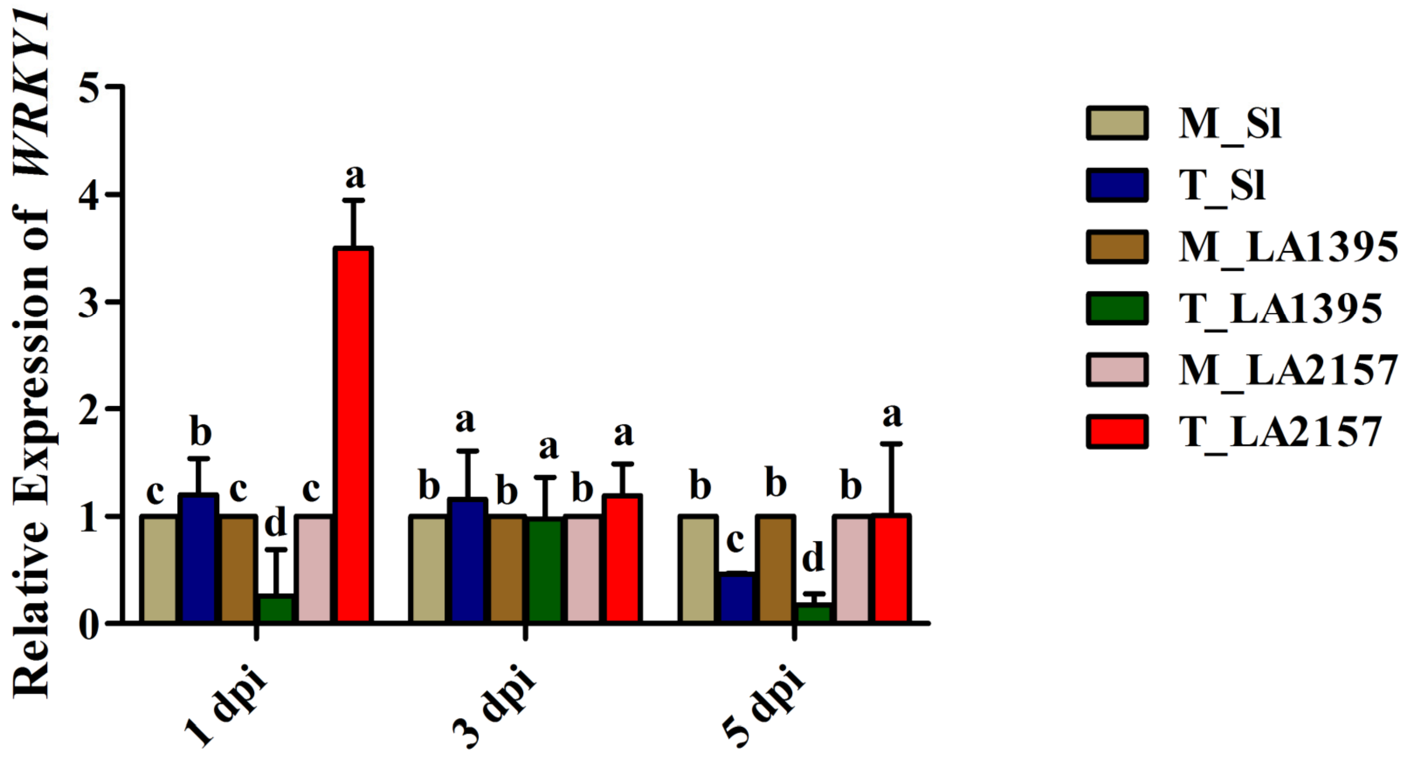

Figure S6 : Expression profiles of *WRKY1* in *S. lycopersicum* (Sl), susceptible (LA1395) and resistant (LA2157) *S. arcanum* accessions. Inoculated with *A. solani* spore suspension (3-4x10^3 spores/mL), leaf samples were collected at 1 dpi, 3 dpi and 5 dpi. qRT-PCR was carried out with *SlEF1α* gene as internal control and expression was normalized to the corresponding *A. solani* spore inoculated (T) and mock inoculated (M) samples. The values represent means ± SE of three biological replicates each with three technical replicates. Bars represent the standard errors of the means. Different letters indicate significant differences according to Duncan's test (P <0.05). Similar pattern of gene expression was obtained in two independent experiments. dpi- Days Post Inoculation, M-Mock inoculated, T- inoculated with *A. solani* spores, Sa- *Solanum arcanum* Peralta.

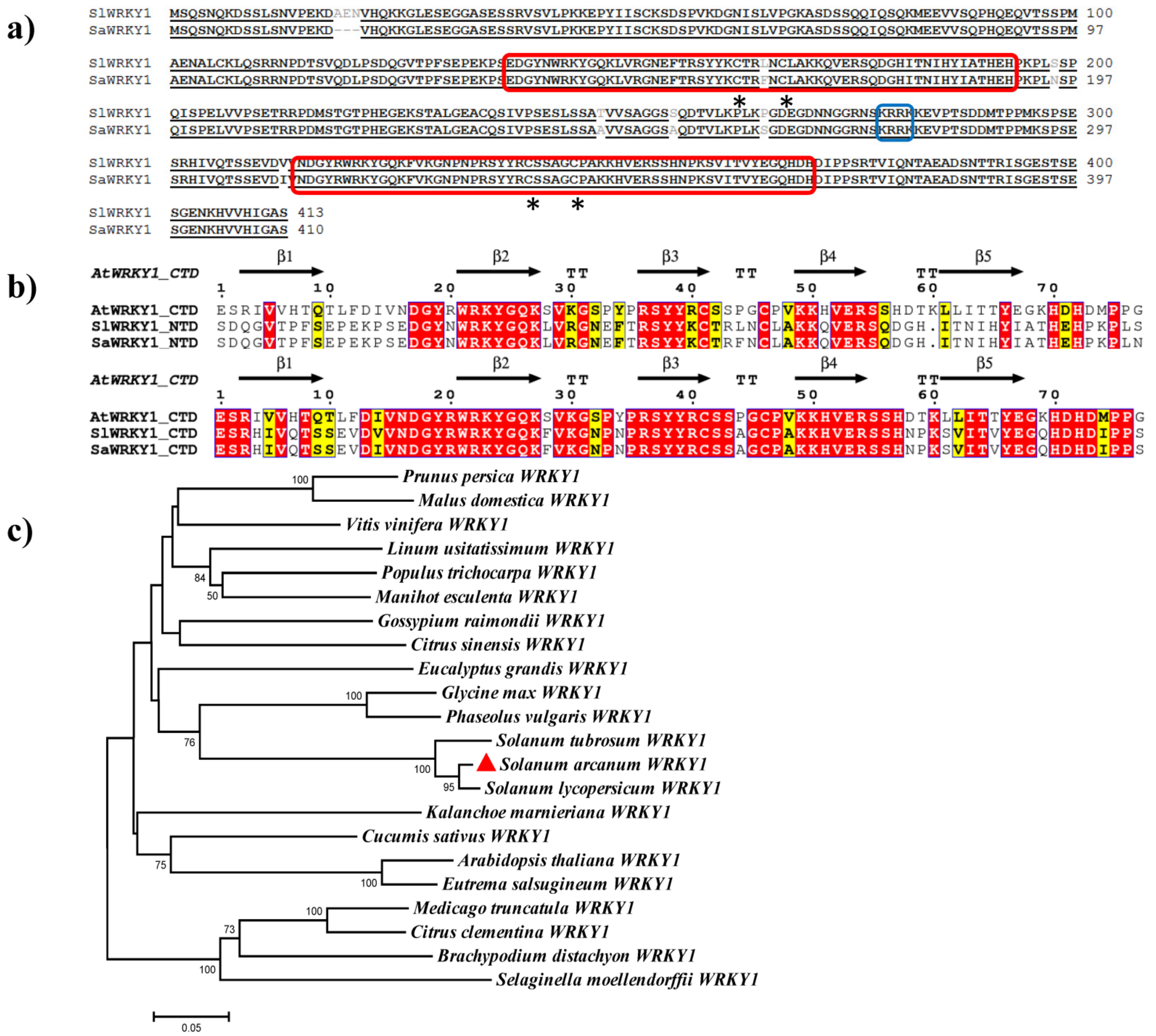

Figure S7. Protein sequence alignment of SaWRKY1 and SlWRKY1 (a). WRKY domains are marked with red box, nuclear localization sequence with blue box and * indicates conserved Cys residues. Structure based sequence alignment of SaWRKY1 C and N terminal domains with tomato and Arabidopsis WRKY1 (b). The secondary structure elements above the sequence blocks correspond to the reported crystal structure of Arabidopsis WRKY1 (PDB ID: 2ayd). Conserved residues are boxed in red. Similar residues are in black bold and boxed in yellow. β-sheets are rendered as arrows and strict β-turns as TT. Phylogenetic analysis of *WRKY1* using amino acid sequences from 21 plant homologs (c) (GenBank IDs. *Solanum arcanum* (KU674828), *Solanum lycopersicum* (Solyc07g047960), *Solanum tuberosum* (PGSC0003DMT400015754), *Arabidopsis thaliana* (At2g04880), *Brachypodium distachyon* (Bradi1g23340), *Vitis vinifera* (GSVIVT01030046001), *Glycine max* (Glyma.02G306300.1), *Gossypium raimondii* (Gorai.007G167100.2), *Populus trichocarpa* (Potri.014G164300.1), *Cucumis sativus* (Cucsa.100440.4), *Manihot esculenta* (Manes.12G062100.1), *Citrus sinensis* (orange1.1g011340m), *Prunus persica* (Prupe.3G202000.4), *Linum usitatissimum* (Lus10012322), *Phaseolus vulgaris* (Phvul.008G286100.1), *Eucalyptus grandis* (Eucgr.A01053.1), *Kalanchoe marnieriana* (Kalax.0043s0092.1), *Malus domestica* (MDP0000256105), *Eutrema salsugineum* (Thhalv10002517m), *Citrus clementina* (Ciclev10007882m), *Medicago truncatula* (Medtr3g056100.5) and *Selaginella moellendorffii* (24407) as an out group) using NJ method with bootstrap value of 1000 replicates.

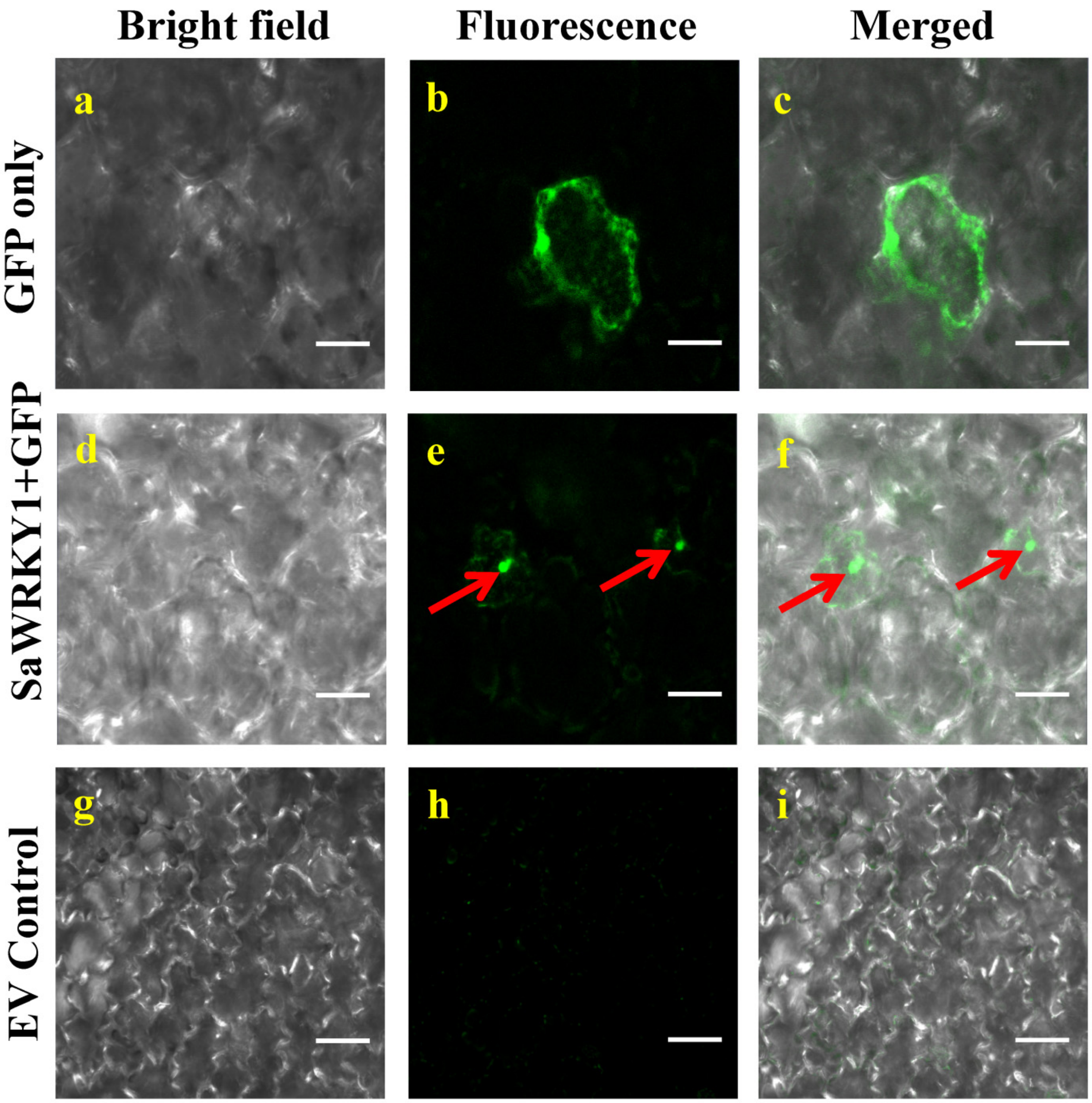

Figure S8. Subcellular localization of GFP in absence and presence of *SaWRKY1* visualized in agro-infiltrated tobacco epidermal cells. Images of agro-infiltrated tobacco epidermal cells using constructs *pRI101-AN:GFP* (a-c), *pRI101-AN:SaWRKY1:GFP* (d-f) and only *pRI101-AN* (g-i) were taken at 6 dpi. All images were taken in a single optical plane. Arrowhead indicates nucleus. Bar represents 100 µm distance.

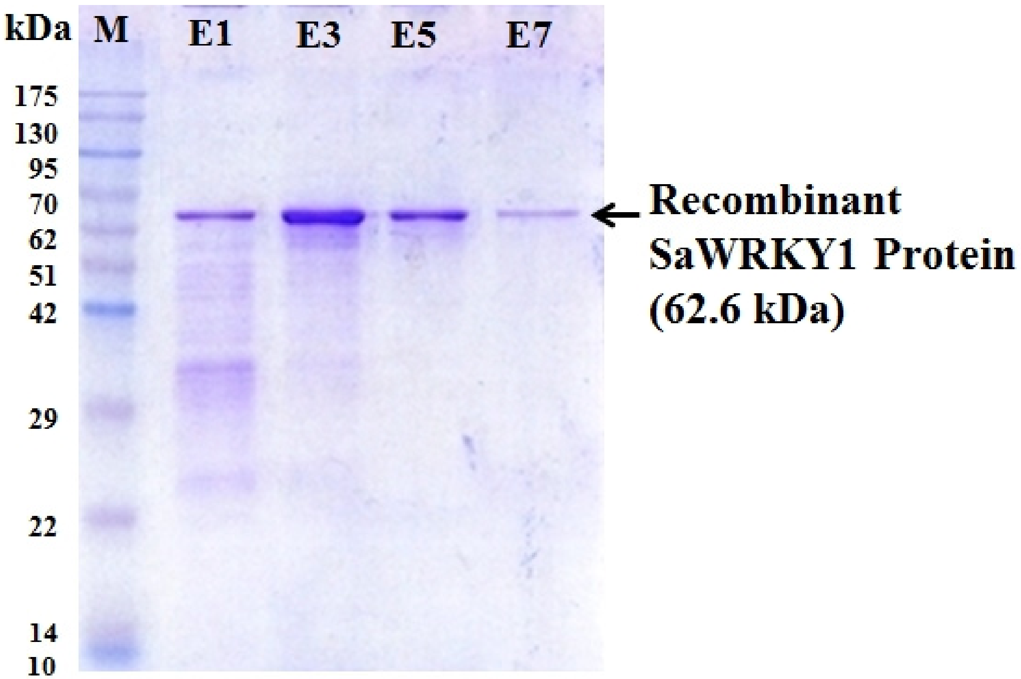

Figure S9. SDS- PAGE (12%) showing purified fractions of recombinant SaWRKY1 protein. M- Standard protein molecular weight marker, E1 to E7- Purified elution fractions.

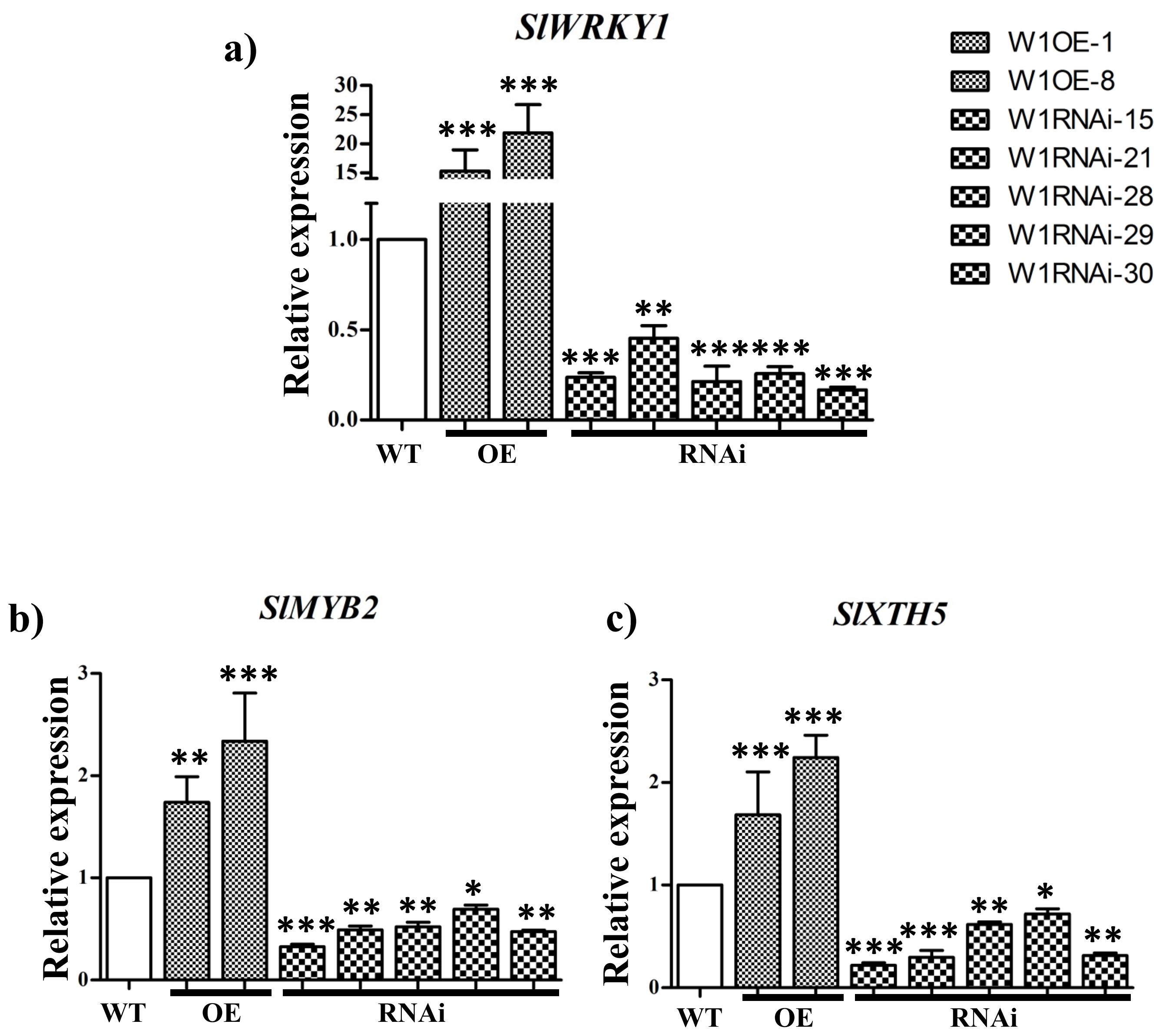

Figure S10. Expression analysis of *SlWRKY1, SlXTH5* and *SlMYB2* using qRT-PCR in T0 transgenic tomato lines without pathogen. Transcript levels of *SlWRKY1* (a)*, SlXTH5* (b) and *SlMYB2* (c) in transgenic tomato lines (*SlWRKY1* OE:W1OE1, W1OE8 and *SlWRKY1* silenced lines: W1RNAi15, W1RNAi21, W1RNAi28, W1RNAi29, W1RNAi30) *SlEF1α* gene was used as an internal control, WT: wild type as non-transformed. W1: WRKY1 In statistical analysis, One-way ANOVA was performed followed by Tukey's post hoc test. Statistical data is significant at *P*-value * $P < 0.05$, ** $P < 0.01$,*** $P < 0.001$.

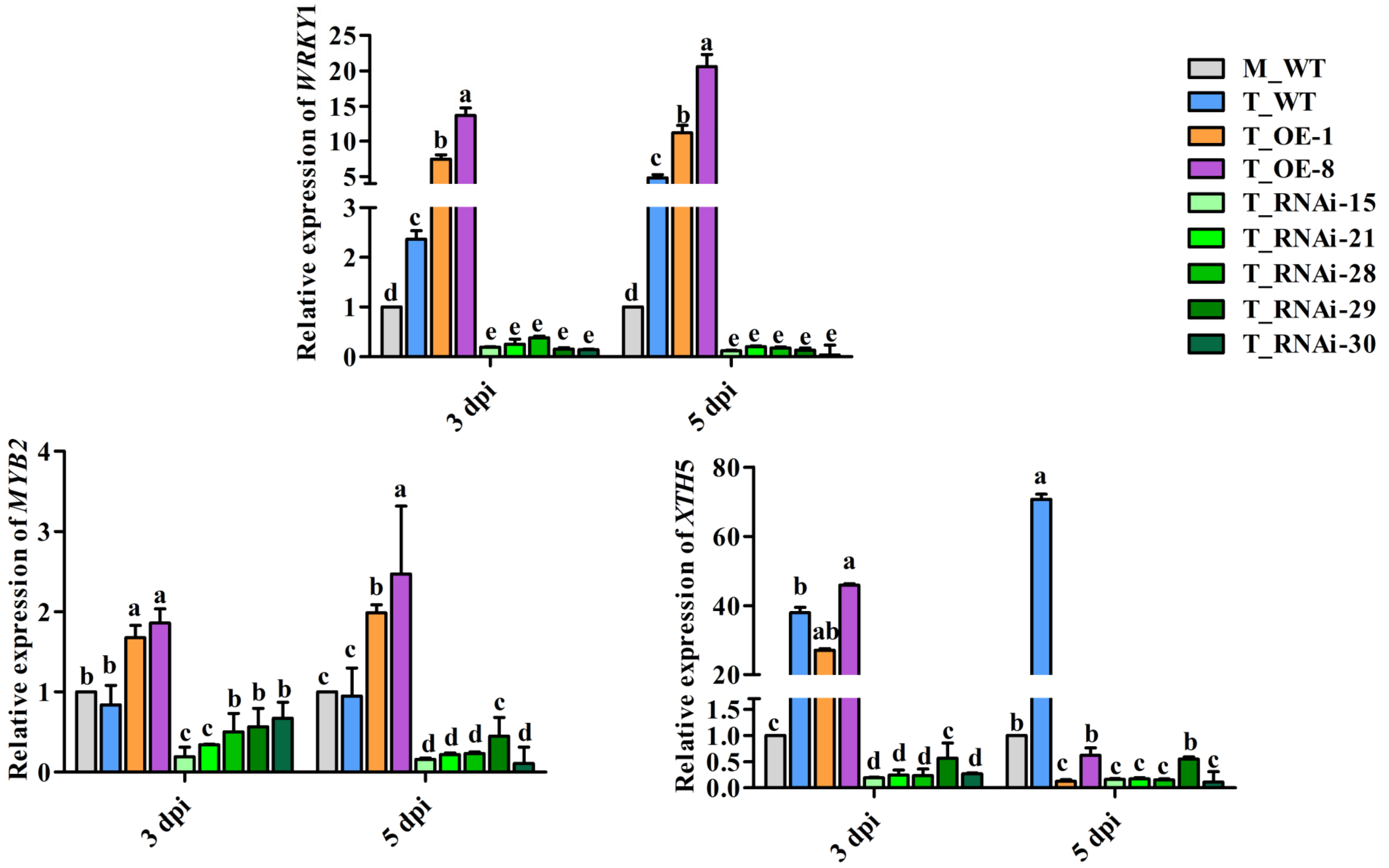

Figure S11. Expression analysis of *SlWRKY1, SlXTH5* and *SlMYB2* using qRT-PCR in T1 transgenic tomato W1OE lines (W1OE-1, W1OE-8) and T0 transgenic tomato W1RNAi lines (W1RNAi-15, W1RNAi-21, W1RNAi-28, W1RNAi-29, W1RNAi-30). qRT-PCR was carried out with *SlEF1α* gene as internal control and expression was normalized to the corresponding *A. solani* spore (T) and mock inoculated (M) samples. Expression variation represents fold change against expression in mock treated WT plants. The values represent means ± SE of three biological replicates each with three technical replicates. Bars represent the standard errors of the means. Different letters indicate significant differences according to Duncan's test ($P < 0.05$). WT: wild type as non-transformed.

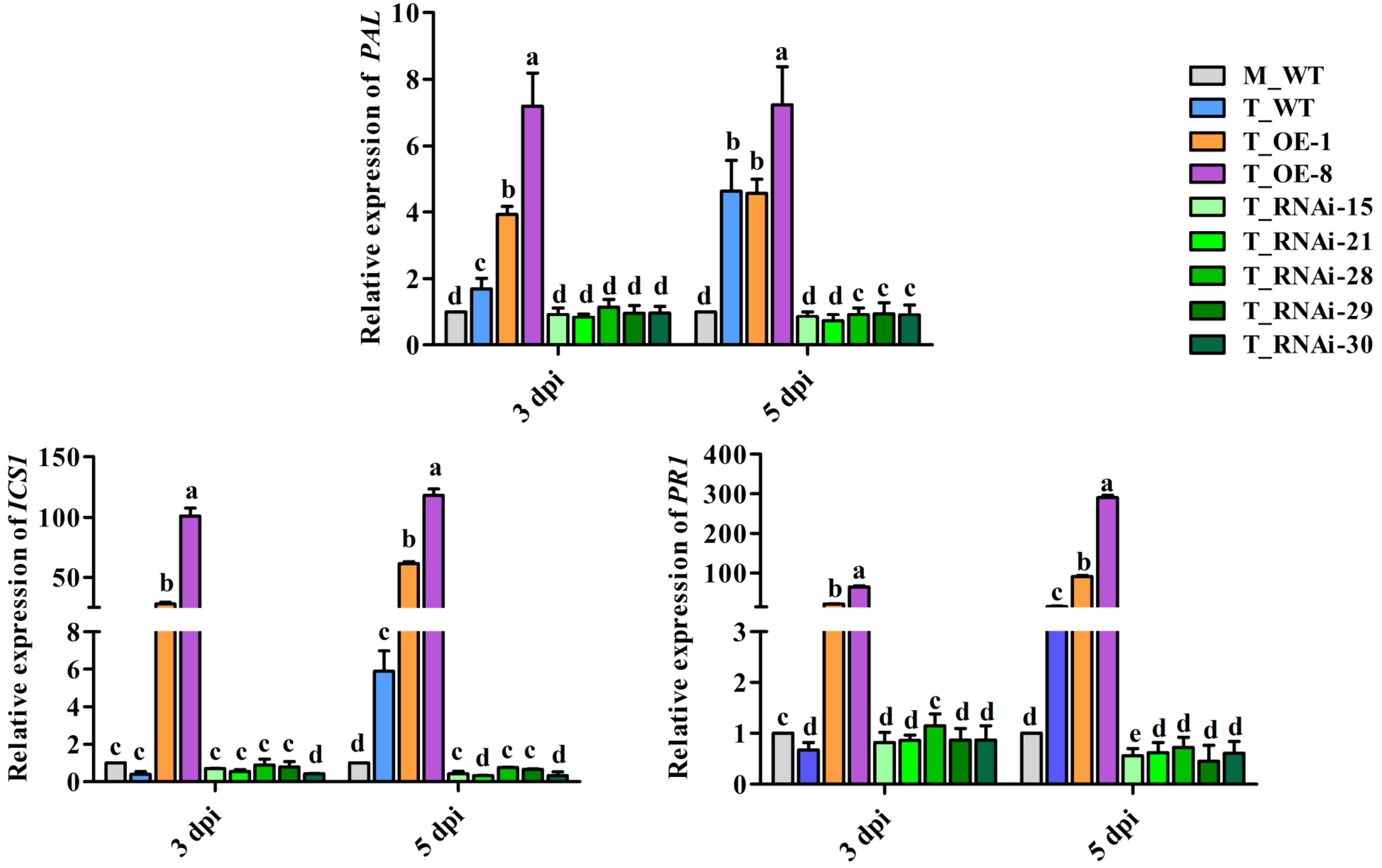

Figure S12. Expression analysis of key genes involved in SA mediated defence response (*SlPAL, SlICS*1 and *SlPR*1) using qRT-PCR in transgenic tomato W1OE lines (W1OE-1, W1OE-8) and T0 transgenic tomato W1RNAi lines (W1RNAi-15, W1RNAi-21, W1RNAi-28, W1RNAi-29, W1RNAi-30). qRT-PCR was carried out with SlEF1α gene as internal control and expression was normalized to the corresponding *A. solani* spore inoculated (T) and mock inoculated (M) samples. Expression variation represents fold change against expression in mock treated WT plants. The values represent means ± SE of three biological replicates each with three technical replicates. Bars represent the standard errors of the means. Different letters indicate significant differences according to Duncan's test ($P < 0.05$). WT: wild type as non-transformed.

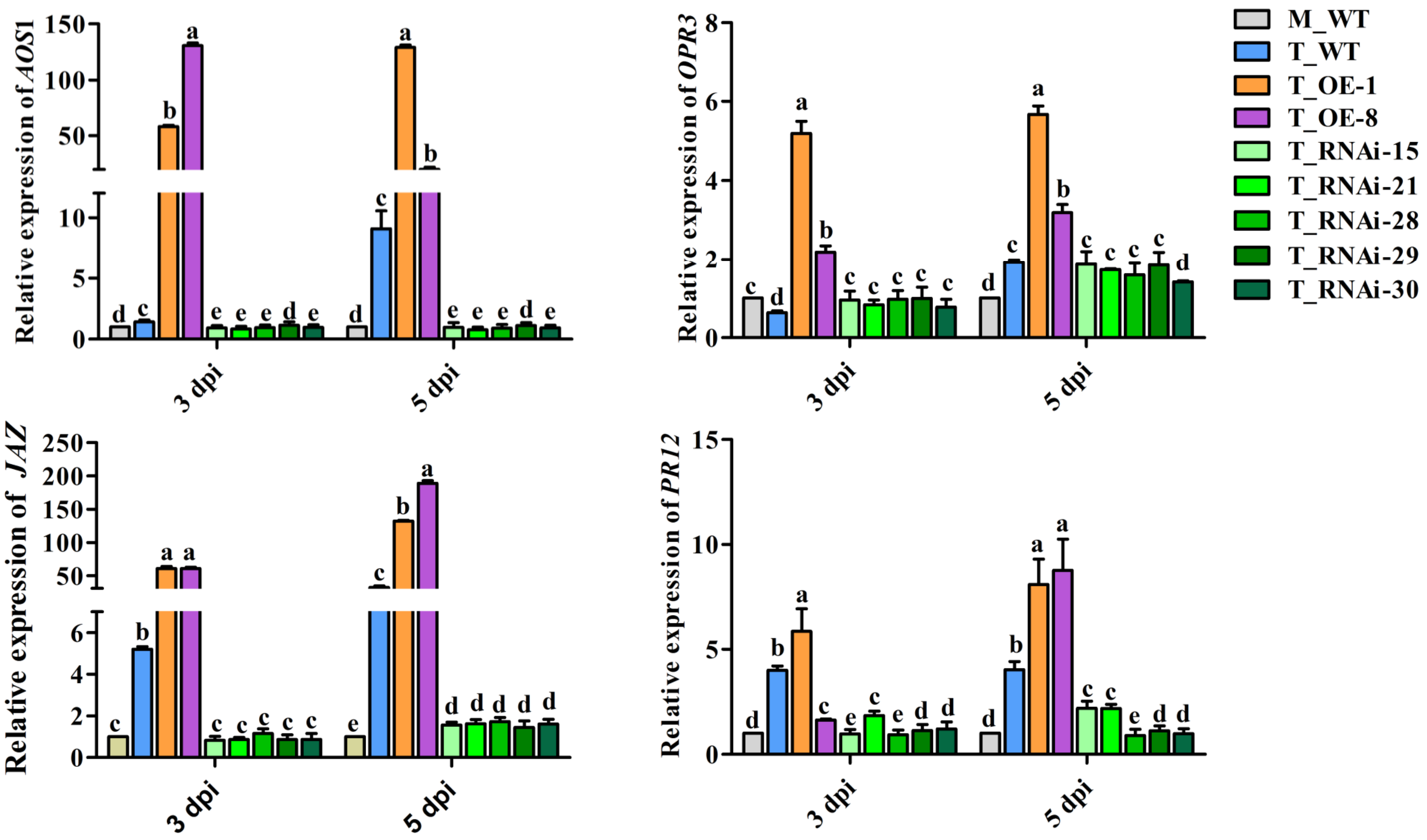

Figure S13. Expression analysis of key genes involved in JA mediated defence response (*SlAOS, SlOPR3, SlJAZ* and *SlPR*12) using qRT-PCR in transgenic tomato W1OE lines (W1OE-1, W1OE-8) and T0 transgenic tomato W1RNAi lines (W1RNAi-15, W1RNAi-21, W1RNAi-28, W1RNAi-29, W1RNAi-30). qRT-PCR was carried out with SlEF1α gene as internal control and expression was normalized to the corresponding *A. solani* spore inoculated (T) and mock inoculated (M) samples. Expression variation represents fold change against expression in mock treated WT plants. The values represent means ± SE of three biological replicates each with three technical replicates. Bars represent the standard errors of the means. Different letters indicate significant differences according to Duncan's test ($P < 0.05$). WT: wild type as non-transformed.
